# Community empowerment and involvement of female sex workers in targeted sexual and reproductive health interventions in Africa: a systematic review

**DOI:** 10.1186/1744-8603-10-47

**Published:** 2014-06-10

**Authors:** Lizzie Moore, Matthew F Chersich, Richard Steen, Sushena Reza-Paul, Ashar Dhana, Bea Vuylsteke, Yves Lafort, Fiona Scorgie

**Affiliations:** 1 MatCH (Maternal, Adolescent and Child Health), Department of Obstetrics and Gynaecology, Faculty of Health Sciences, University of the Witwatersrand, Durban, South Africa; 2 Centre for Health Policy, School of Public Health, University of the Witwatersrand, Johannesburg, South Africa; 3 International Centre for Reproductive Health, Department of Obstetrics and Gynaecology, Ghent University, Ghent, Belgium; 4 Wits Reproductive Health and HIV Research Unit, Faculty of Health Sciences, University of Witwatersrand, Johannesburg, South Africa; 5 Department of Public Health, Erasmus MC University Medical Centre, Rotterdam, The Netherlands; 6 Community Health Sciences, University of Manitoba, Manitoba, Canada; 7 Institute of Tropical Medicine, Antwerp, Belgium

**Keywords:** Female sex workers, Community mobilisation, Structural interventions, Africa

## Abstract

**Background:**

Female sex workers (FSWs) experience high levels of sexual and reproductive health (SRH) morbidity, violence and discrimination. Successful SRH interventions for FSWs in India and elsewhere have long prioritised community mobilisation and structural interventions, yet little is known about similar approaches in African settings. We systematically reviewed community empowerment processes within FSW SRH projects in Africa, and assessed them using a framework developed by Ashodaya, an Indian sex worker organisation.

**Methods:**

In November 2012 we searched Medline and Web of Science for studies of FSW health services in Africa, and consulted experts and websites of international organisations. Titles and abstracts were screened to identify studies describing relevant services, using a broad definition of empowerment. Data were extracted on service-delivery models and degree of FSW involvement, and analysed with reference to a four-stage framework developed by Ashodaya. This conceptualises community empowerment as progressing from (1) initial engagement with the sex worker community, to (2) community involvement in targeted activities, to (3) ownership, and finally, (4) sustainability of action beyond the community.

**Results:**

Of 5413 articles screened, 129 were included, describing 42 projects. Targeted services in FSW ‘hotspots’ were generally isolated and limited in coverage and scope, mostly offering only free condoms and STI treatment. Many services were provided as part of research activities and offered via a clinic with associated community outreach. Empowerment processes were usually limited to peer-education (stage 2 of framework). Community mobilisation as an activity in its own right was rarely documented and while most projects successfully engaged communities, few progressed to involvement, community ownership or sustainability. Only a few interventions had evolved to facilitate collective action through formal democratic structures (stage 3). These reported improved sexual negotiating power and community solidarity, and positive behavioural and clinical outcomes. Sustainability of many projects was weakened by disunity within transient communities, variable commitment of programmers, low human resource capacity and general resource limitations.

**Conclusions:**

Most FSW SRH projects in Africa implemented participatory processes consistent with only the earliest stages of community empowerment, although isolated projects demonstrate proof of concept for successful empowerment interventions in African settings.

## Introduction

Sex workers are highly vulnerable to health and social problems, including sexually-transmitted infections (STIs), unintended pregnancy, violence, exploitation, discrimination and substance abuse [1]. For example, in countries with generalised HIV epidemics, the odds of a female sex worker (FSWs) to be living with HIV is 13.5 times that of other women [2]. Social marginalisation and a typically criminalised working environment limit the ability of FSWs to mitigate the impact of their occupational hazards, such as multiple sexual partners, difficulties in negotiating condom use, poor access to appropriate lubricants and high STI prevalence [3,4]. Up to 4.3% of women and girls engage in sex work in sub-Saharan Africa [5], yet FSW interventions in the region have historically operated in isolation with limited national or international support, in strong contrast to larger coordinated sex work projects in other settings [6]. Despite being a priority population for HIV interventions globally, coverage of prevention, treatment and support programmes among FSWs in the region remains low [7,8].

International discourse on the health of vulnerable populations has increasingly favoured ‘structural interventions’ – in other words, interventions addressing ‘macro-level’ structural forces that *“shape distributions of power within and across societies”*[9] – over those targeting bio-behavioural risk at the individual level [9-12]. In India, targeted FSW interventions have for long adopted a community-centred approach, with a particular focus on transforming politico-legal or socio-cultural structures [13-15]. In the early 1990s, the Sonagachi Project in West Bengal, India implemented one of the first developing country models of sex worker services that incorporated community and societal-level theories of change into HIV/STI prevention interventions [13]. As a measure of its success, the community-based organisation Durbar Mahila Samanwaya Committee (DMSC), which formed in 1995 and has a current membership of 65 000 sex workers, has been running the Sonagachi Project since 1999. Other sex worker-led organisations such as Ashodaya Samithi in Mysore (hereafter ‘Ashodaya’) have integrated the Sonagachi approach into broader community-led interventions to reduce violence and the structural underpinnings of HIV vulnerability. This model has since been rolled out across six states as part of the Avahan India AIDS Initiative [14]. Such projects have arguably achieved greater impact, in terms of clinical and social outcomes, coverage and sustainability, than those which seek to build individual responsibility for effective behaviour change [15,16].

Lessons drawn from several large evaluations of Indian community empowerment models [13,15,17,18] suggest that these models lend themselves to adaptation in the African context. However, much remains unknown about whether FSW community empowerment initiatives have been implemented in Africa or, indeed, whether they have the potential to make as impressive an impact as they have done in India. It is also unclear how interventions using this broad approach to empower sex worker communities in African settings have been structured. To date, health services across the world have primarily engaged with FSWs through provision of sexual and reproductive health (SRH) services, often in the form of targeted interventions designed to interrupt transmission of HIV and STIs between FSWs, their clients and the wider population [19]. This article presents findings from a systematic review of SRH interventions targeting FSWs in Africa, focusing on projects that include elements designed to empower communities. Firstly the review aims to describe the nature and structure of targeted SRH interventions encompassing empowerment processes. Thereafter, the review evaluates the progression of ‘community empowerment’ interventions in Africa from the perspective of SRH service provision – in other words, the extent to which SRH interventions have provided an enabling environment for community mobilisation and structural change. For this assessment, we use a four-stage framework of community empowerment devised by Ashodaya and DMSC in India (examples are primarily drawn from Ashodaya). This is a sub-study of a larger systematic review of targeted SRH service delivery models across Africa and India, undertaken for the DIFFER project (Diagonal Interventions to Fast-Forward Enhanced Reproductive health).

## Conceptual framework

The most recent WHO guidelines on HIV and STI prevention and treatment for sex workers (2012) recommend that all health services, including primary health care, are made *‘available, accessible and acceptable to sex workers based on the principles of avoidance of stigma, non-discrimination and the right to health’*[19]. Critically, these guidelines consider a package of interventions to enhance community empowerment among sex workers *“absolutely necessary”* for improving living and working conditions and redressing human rights violations. In the context of formulating a set of recommendations for such interventions, the WHO defines community empowerment as “*a collective process through which the structural constraints to health, human rights and well-being are addressed by sex workers to create social and behavioural changes, and access to health services to reduce the risk of acquiring HIV*” [19]. We use this definition throughout the article, since it approximates most closely the way ‘community empowerment’ is operationalised by Ashodaya (which, to our knowledge, has not explicitly developed a definition of its own).

In the academic literature it is generally considered more insightful to define empowerment as a process rather than an outcome [20,21], or as an interaction between both process and outcome [22,23]. Laverack’s conceptualisation of community empowerment as a ‘dynamic continuum’ from individual action to collective social and political change [20] is most consistent with Ashodaya’s approach. In this formulation, components of an empowerment intervention should be designed to enable people to maximise their potential to progress along this continuum [20]. Fundamentally, Ashodaya aims to respond to the needs of the community by encouraging community mobilisation and critical consciousness-raising among sex workers, to enable transformation of the power relations that shape their worlds. In this respect, the approach draws on the tradition of ‘liberatory pedagogy’ and notions of collective empowerment as advanced by Paolo Friere and others [24].

For example, after identifying the ‘governance’ of sex work and its policing as a key community concern, Ashodaya’s advocacy efforts to reduce vulnerability in this area included holding ‘sensitisation’ meetings with local police officials and sex workers. These meetings challenged popularly held stigmatised notions of sex workers and highlighted how police could change their attitudes towards sex workers and assist in HIV prevention. Collectively, sex workers were empowered to confront police action they considered improper, through provision of legal literacy, support from a 24-hour crisis intervention outreach team and nurturing a culture of rights. These efforts resulted in a marked reduction in violence from police, clients, local thugs and shopkeepers, and cultivated an image of sex workers as active agents for social change [15]. In short, the community’s response to violence and their progressive empowerment featured mobilising the sex worker community, collectively designing an intervention and ensuring community ownership of it, thereby shifting structural vulnerabilities in the long term.

**Figure 1:**
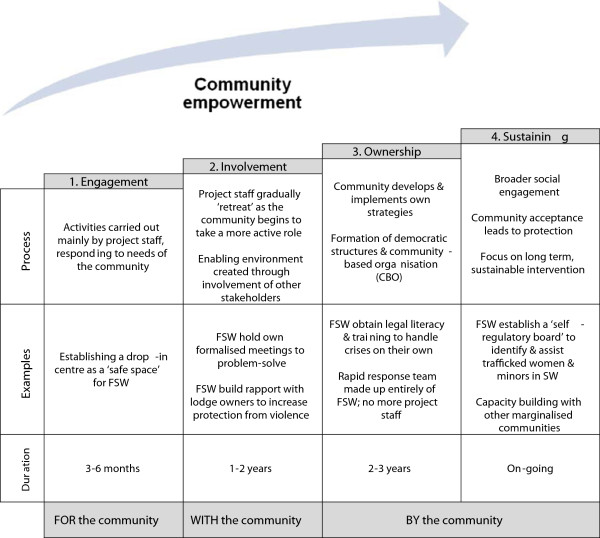
**Stages of empowerment in Ashodaya’s model of implementing community-based structural interventions (adapted from Reza-Paul *et al., *2012)**[14].

Ashodaya applies a four-stage framework to conceptualise the progression of empowerment in the context of their efforts to reduce violence and HIV risk: (1) engagement with the sex worker community, (2) involvement of the community in targeted activities, (3) ownership of the project by the community (4) sustainability of action beyond the community (Figure 1) [15]. Ashodaya’s framework is useful insofar as it provides a means of analysing the complex relationship between empowerment and the challenge of confronting stigma, discrimination and gender-based violence [25].

To assess FSW community empowerment in African settings, this paper considers the extent to which projects undertake activities that facilitate the *processes* defining each stage of this framework. Focus is not placed on individual examples, since these are likely to be context-specific. Our aim is not to compare African and Indian interventions *per se*, much less to suggest that the Indian trajectory is necessarily the ‘gold standard’ that other regions should uncritically emulate. Rather, the framework is useful merely as a heuristic device to illuminate the progress made by African service delivery models from an empowerment perspective.

## Methods

On 22 November 2012, we searched Medline (Pubmed interface) and Web of Science, without date restrictions, for studies of SRH service provision to FSWs in Africa. Search terms used in Medline were: “prostit*” or “sex work” or “sex worker” or “sex workers”, and all low- and middle-income countries [26] (MeSH term or any field). Articles were located in Web of Science using the terms “sex work” or “prostitution”, and then filtered to include only African countries. The full search strategy is available on request. Some experts in the field were contacted for additional service models, but we did not aim to comprehensively identify projects not reported in the peer-reviewed literature. Authors of articles were not contacted for additional information.

Titles and abstracts were screened using EPPI-Reviewer (United Kingdom, version 4) [27]. Duplicate references were removed, and abstracts and titles then screened using pre-specified inclusion and exclusion criteria. Full-text articles were reviewed where inclusion could not be established on review of title and abstract alone. Review methods follow the PRISMA guidelines [28], however, because the review examined programme design and not intervention outcomes, some modification was required, such as assessment of risk of bias. The EPPI-Reviewer 4 software and Endnote were used to index all documents located and later referenced.

### Inclusion and exclusion criteria

Included in this review were all projects that provided SRH services (such as those addressing STIs, including HIV, family planning, safe abortion, sexual behaviour and gender-based violence) and which also encompassed processes consistent with the WHO definition of community empowerment. Based on the assumption that the vast majority of health service interventions targeting FSWs include SRH services, we believe this inclusion criterion covers studies within the wider health service domain. Although we do acknowledge the high probability that several important sex worker empowerment interventions exist outside of the parameters of health service provision, these projects do not fall within the scope of this research. FSWs were defined as women who receive money or goods in exchange for sexual services, either regularly or occasionally [7]. Both qualitative and quantitative studies were included, as well as articles providing only a description of a sex worker programme. Further, for maximum inclusivity, the review included targeted, stand-alone projects serving this population exclusively or in addition to other groups, and FSW interventions couched within general population services. Services could be facility-based or provided in the physical spaces within which FSWs live or work (i.e. ‘sex work hotspots’) and could be initiated within the private or public sector. Finally, we also included sites set up primarily for research purposes, but which nonetheless extended clinical services to FSW participants. Studies of services that were provided exclusively for similar but distinct high-risk groups, such as female bar workers and clients or partners of FSWs were excluded, as were studies that only described characteristics of the FSW population or its needs. Studies in languages other than English were excluded, as were studies based solely on mathematical modelling of interventions.

### Study variables and data analysis

Duplicate data extraction was not done. Those doing extractions were trained, their work reviewed and feedback provided. During extraction, queries were noted which were then discussed with other team members and resolved by consensus. Information from full-text articles was extracted according to the following variables: target groups; the physical setting in which services were provided (such as a clinic or other facility, or home- or work-based outreach); whether the intervention was ‘community-based’ (meaning that services were either provided within geographical areas where FSWs are known to live and work or those which incorporated activities responsive to community needs and extended beyond the walls of the clinic); clinical, health promotion and any other services offered; the extent to which FSWs themselves were involved in or led service delivery; and types of human resources responsible for service delivery. Finally where these existed, details of evaluations of interventions and changes to service packages over time were noted. To address the two study objectives, the data extracted for each variable were examined to identify themes emerging in the data. We then synthesized all findings from the individual variables to form an overall analysis and identify trends. Unique illustrative components were also noted.

## Results

**Figure 2:**
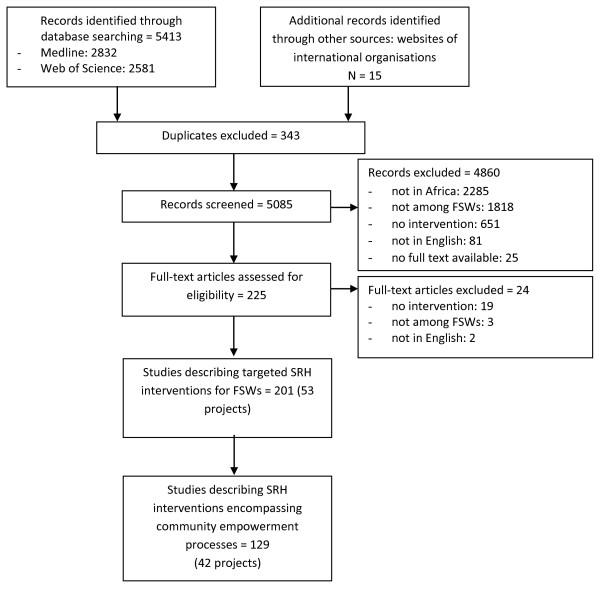
Flow chart of identification and selection of studies.

Of the 5413 articles screened, 129 were included, describing 42 different projects incorporating elements of FSWs community empowerment in 26 different African countries (Figure 2 and Additional file 1: Table 1).

Results are presented in two sections: the first describes the range of service delivery models and the second assesses the overall progression of community empowerment in African FSW interventions, using the conceptual framework detailed above.

### Service delivery models

1. *Target groups*

Most interventions provided services exclusively for FSWs, though some broadened their priority population to include male and transgender sex workers [29-41]. It was often unclear whether other vulnerable groups aside from FSWs were also permitted or encouraged to access services. In some cases, specific FSW target sub-groups were identified, such as those who were brothel-based [42], street-based [41] or bar-registered [43,44]. Women who engaged in occasional sex work, but who depended primarily on other means of income generation [45-51] and other ‘high-risk women’ [52-55] were also prioritised by several projects, demonstrating understanding of the broad spectrum of transactional sex that characterises many African settings. Male partners of FSWs [40,48,56-66] and (potential) male clients [67-69] – including truck drivers [70,71], mine workers [72] or military personnel [73,74] – were frequently targeted in HIV prevention interventions alongside FSWs.

2. *Project settings and service packages offered*

The general pattern of service delivery was via small, isolated projects with limited coverage, or within the context of large research studies. About two thirds of projects provided clinical and health promotion services in a primary health care or STI clinic setting, which then acted as a base for associated outreach. The remainder worked exclusively in the community. To optimise access, almost all clinic-based projects established dedicated, targeted centres inside or within close proximity to areas in which FSWs live and work [45-50,60,75-99]. A sub-category of these community-based, targeted interventions were situated at truck stops along major transport routes [40,52,70,71,100-109]. Although not always clear, it seems a very small minority of projects eschewed the model of targeted clinics altogether, and instead provided an integrated service package for FSWs within general population health services [42,110-125].

In most cases, clinical services were largely delivered within medical facilities and tended to focus on condom distribution and free STI and HIV prevention interventions, such as periodic presumptive treatment of STIs and HIV counselling and testing. The specific range of these services is described elsewhere and thus is not covered in detail here. We identified only five projects that additionally provided general primary health care services [29-34,41,68,88-99,126-130]. Strikingly few provided broader SRH services: only two reported family planning services [109,131] and two others HIV treatment, care and support [132,133]. None offered counselling for unintended pregnancy or termination of pregnancy services, cervical cancer or gender-based violence, although one study mentioned referral pathways [134].

A variety of settings and modes of community outreach was identified. Health promoters, community health workers, peer educators and other field staff visited individual FSWs in their homes [132,133,135], or held group or individual sessions within brothels [35-40], hotels [136] and places of entertainment [73,74,137-139]. Although a wide range of outreach activities was undertaken across all the projects, again most appeared to be geared towards HIV and STI prevention through individual behaviour change. Thus outreach services most frequently encompassed STI and HIV prevention education, condom distribution and promotion and recruitment for STI screening [40,48,56-66,83,99,110-121], often via a referral card system [52,55,140,141]. Most projects encouraged FSWs to ‘drop in’ to a clinic to collect free condoms; however we identified only three projects (in Ghana, Mombasa Kenya, and The Sex Worker Education and Advocacy Taskforce (SWEAT) in Cape Town South Africa) which had established an actual community drop-in centre that could serve as a meeting place and central hub for distribution of condoms and information, education and communication (IEC) materials [41,60,75-80,142-147]. Finally, clinical service provision via outreach was uncommon: mobile units providing STI and other services to FSWs were documented in only five projects, all in Southern Africa [35-40,43,44,55,72,134,140,141].

3. *Approaches to community empowerment and degree of sex worker involvement*

a. FSW peer education

Peer education was the primary empowerment approach adopted by the vast majority of projects reviewed. Peer educators – either current or former sex workers – would visit FSWs in their homes, places of work or at community drop-in centres, engaging with them either individually or in group meeting formats. In most cases, peer education programmes were linked to a specific clinic facility, from which training was coordinated and supplies obtained. Referrals for STI screening and treatment or for other services such as HIV counselling and testing were then also made to this facility.

The package of services provided by peer workers varied markedly across projects, and included: recruitment or encouragement of FSWs to visit a clinic for STI screening; guiding FSWs to the clinic; diagnosis and treatment; education in HIV and STI prevention and in condom negotiation skills; condom demonstration, promotion and distribution; general health education; referrals for clinical services; risk-reduction education or counselling; distribution of IEC materials and safer-sex kits; and awareness-raising of available clinical services. Only one project based along the Trans-Africa highway in Kenya and Uganda offered peer-based family planning services to sex workers [109] and only SWEAT in Cape Town provided legal assistance to sex workers who had experienced violence and assault [41]. Unconventional participatory methods such as drama [73,74,134,142-147] and art [88-98] were implemented by four projects.

Peer education projects typically encompassed non-FSW personnel, such as community health workers or field workers, who formed close working relationships with peer educators, undertook training and linked them to the facility and its clinical personnel [35-40]. Although contact with the wider FSW community was mainly through peer educators, a minority of projects employed research staff or field workers specifically to approach FSWs and male clients [40,41,48,56-66,82,131]. Some field coordinators trained peer educators [45-50,52] or supervised and monitored peer educators activities [142-147]. Projects frequently employed their own clinical staff and counsellors [60,68,75-80,83,110-121,126-130], and health promoters [35-40,137]; one study specifically mentioned employment of monitoring and evaluation specialists [133]. We identified five projects designed to reduce workplace vulnerability by involving other players within the sex industry, and those on its fringes, as peer educators. These included: non-paying partners [132], male clients [40,48,56-66], bar, security and sales personnel in entertainment venues [68,69], female bar and guesthouse workers, male petrol station workers and social welfare and transport officers [52]. Generally project hierarchy dictated that FSWs involved in the project would report to non-FSW personnel who occupied supervisory or management positions; few exceptions to this rule are discussed below.

b. Involving FSWs beyond peer education

The second most common mode of FSW involvement was consultation on project design, implementation and/or management. This was usually implemented within the peer education model [55,68,71,110-121,126-130,136,140,141]; only three projects appeared to use community consultation as the sole empowerment process [42,67,148,149]. The level of formality assigned to community consultation varied: in one Kenyan site (Mukuru, Nairobi) FSWs were simply invited to presentations about the research project during planning stages [67], whereas in one Madagascan site FSW representatives presented their perspectives on study design during a three day workshop prior to commencing research activities [110-121] and in another Madagascan site were actively involved in drawing up project guidelines [150,151]. A more continuous formal consultation process was developed in the Pumwani Majengo project in Nairobi, Kenya (the Majengo slum makes up part of the Pumwani District), whereby individual FSWs were elected by their peers as committee members to oversee research, decide priorities and communicate with researchers [68,126-130]. In Virginia, South Africa, peer educators met weekly with the clinic nurse to plan activities and discuss solutions to problems encountered [55,140,141]. Similarly, in Cross River State, Nigeria, monthly meetings were held with elected ‘Chairladies’ to elicit input into project implementation and serve as a forum to discuss topics of concern [136].

A few projects undertook to provide FSWs with skills allowing them to adopt roles of greater responsibility within or outside of the organisation. For example, in Bulawayo, Zimbabwe ‘senior peer educators’ were trained to organise, motivate and supervise a cadre of peer educators within a geographic zone [68,69]. In Rwanda, ‘community-mobilisers’ (prominent community members with extensive social networks) were encouraged to lead project meetings [84-87]. FSW peer educators in Togo were trained to map sites and enumerate the FSW population [82]. Similar activities, using capture-recapture techniques, occurred in Madagascar, Kenya and Côte d’Ivoire [99,151]. We found two cases in South Africa where peer educators were taken on as formal project employees: firstly as a manager in a project targeting sites along the Johannesburg-Durban highway [40,100-108] and secondly as field workers in a research study based at SWEAT in Cape Town [41]. Other FSWs involved in this study volunteered to recruit participants and develop an interview schedule for research. SWEAT also actively involved FSWs in political advocacy, encouraging and supporting them to join health educators in lobbying for the right to better access to public ablution facilities. Moreover, in response to frequent cases of police harassment, FSWs were educated on their legal rights, giving them skills and vocabulary for political advocacy and for assertive communication within their personal lives [41].

c. Financial empowerment

Capacity building for financial empowerment within the context of health service provision was uncommon. ‘Exit programmes’ that provide peer educators skills training in alternative employment have been trialled in South Africa [35-40], and one Malawian study mentioned ‘options for income generation’ as a core component of peer educator training [138]. After requesting support for business activities, FSWs participating in peer-education activities in Pumwani Majengo, Nairobi, were provided with credit for small businesses such as commodity trading and manufacturing, as well as business skills training and mentorship. On-time loan repayment was achieved at 65% and two-thirds of participants sustained their businesses at follow up after 18–23 months, as well as reporting increased condom use with regular partners and a significant reduction in the number of regular sexual partners [126]. Although this venture appears to have been successful, we found no evidence of replication or implementation of similar microfinance projects for FSWs associated with SRH service delivery elsewhere in Africa.

d. Engaging the wider community

Wider community engagement and education was relatively common. Two projects used this as a primary means of reaching FSWs: in Shurugwi, Zimbabwe, project staff held sexual health education sessions in beer halls, community halls, work places and commercial farms, thereby targeting FSWs within a wide audience by default [139]. Similarly in Mukuru, Kenya, community health education and project planning meetings were held with local administrators and village elders, to which FSWs were also invited [67]. Wider community engagement was more commonly used to augment peer-education activities, by attempting to change social norms and address structural causes of social vulnerability: peer education projects in Zambia (the Corridors of Hope project) [134] and West Africa [133] invited members of local and district government, civil society groups and local organisations to participate in committee meetings, in order to provide a forum in which all stakeholders could raise their concerns, and through which positive prevention messages of the project could be spread. The Zambian Corridors of Hope project also worked with ‘Queen Mothers’ (former FSWs employed by landlords to supervise FSWs in guest houses) to raise awareness of the project and facilitate effective implementation [134].

In Nigeria, emphasis was placed on educating ‘gatekeeper’ individuals – those living in FSWs’ immediate environment who provide services, advice or protection and who hold some influence over them, such as police commissioners or local security agents [136]. Similarly in Cape Town, South Africa, pimps – who were highly supportive of the project – were encouraged to accompany FSWs to drop-in centres and monitor and report abuse from clients to SWEAT project staff [41]. This project also engaged directly with clinical staff in general population health services to raise their awareness of the needs of FSWs and the hardships they face, thereby hoping to reduce stigma and discrimination in clinical settings [41]. Finally, in Malawi FSWs were invited to participate in ‘sensitisation sessions’ directed at bar and club owners and disc jockeys working at clubs, thus encouraging interaction among these groups. Disc jockeys then distributed safe sex messages and materials via quizzes and handed out awards to club patrons [138].

### Progression of FSW empowerment in Africa

1. *Engagement: For the community*

Within the Ashodaya framework, community engagement primarily involves responding to the community’s expressed needs and demands. African FSW ‘empowerment’ interventions have generally been successful in establishing strong initial links with their target communities, predominantly using a clinic-based peer-education model. Within this model, community representatives interacted with project staff and inputted into project design and implementation. Communication networks were usually established with FSWs using a snowball technique, following identification of natural FSW leaders by community health staff or key informants. In Madagascar, some FSWs were contacted through a formal association granting them access to the local port to solicit clients, on the condition that they made regular visits to the public STI clinic [150,151]. Similarly, health educators in Senegal were able to contact FSWs by virtue of a legal requirement for them to register with one of four national specialist medical centres [81,152,153]. In Guinea, FSWs were also required to visit the STI clinic on a monthly basis [122,123]. It is unclear whether these prescriptive methodologies were more successful than ‘snowball recruitment’ in gaining bilateral trust for meaningful engagement.

A large number of projects established a community-based STI clinic as a central point for education activities, however projects rarely prioritised forums or physical spaces to promote ongoing communication between project staff and the community. In the Indian example, providing a drop-in centre proved to be an important step in facilitating community engagement; in Ashodya’s case this was promoted as a ‘safe space’ for FSW to congregate in response to the community’s expressed needs [15]. Unlike in India, community engagement through crisis response appears rare in Africa, aside from SWEAT’s work in Cape Town, South Africa, where field workers helped FSWs in the event of arrest or court appearance, and with bail applications [41].

Some studies, particularly those located around transport corridors, commented on obstacles to establishing initial community linkages, such as heterogeneity, disunity and fluxes in the composition of the FSW population [88-98,137]. In other projects, however, FSW community engagement strategies expanded and evolved over time, suggesting that these obstacles are not insurmountable. For example, the observational cohort study group working with highly disenfranchised FSWs in Pumwani Majengo, Nairobi, amongst whom no natural community existed, actively responded to growing awareness of the importance of community engagement and potential channels to achieve this. Through initial one-on-one communication between field workers and FSWs, they were able to build a two-way system of trust and common purpose, which enabled the community to unite around the clinic, with natural leaders linking research staff to the wider FSW community [130].

2. *Involvement: With the community*

Establishing networks of peer educators could be viewed as an initial step towards more formal ‘community organising’. Peer educator projects, however, were seldom designed in such a way that FSWs could begin to take more active roles and ultimately lead the process of collective social or political action. Indeed, there were few instances of peer workers being supported to take on supervisor or coordinator positions. FSW collectivisation appears to have no precedent in Africa, and has consequently been difficult to initiate. One African study (Mombasa Kenya) specifically commented on the importance of their drop-in centre in promoting community involvement; this acted as an important training and meeting facility and in turn enabled peer educators to hold monthly community gatherings with the active participation of FSWs [145]. Yet provision of spaces to enable independent community organising elsewhere was rare.

General community meetings in public spaces have reportedly provided a nurturing environment for formation of FSW leadership groups [68]. In the Zambian Corridors of Hope project, a participatory methodology was applied to community meetings to stimulate dialogue and action within a democratic forum, in order to encourage FSWs to reflect on their problems and unite in collective action [134]. However, it was unclear to what extent such community meetings ultimately enabled the FSWs to proactively define a course of collective action. Equally, in cases where a wide range of stakeholders was consulted on project design [133,134], it is unknown whether this enhanced empowerment, or actually undermined the ability of FSWs to dictate their own requirements.

Lobbying of city authorities by FSWs in Cape Town, South Africa, for a cleaner work environment [41] was the only instance identified of true community-led advocacy, and we found no documented attempts by service providers or FSW groups themselves at major interaction with the police. Although significant, examples of other structural interventions to increase workplace security, such as targeting (potential) male clients [67-70,72], ‘gatekeeper individuals’ [136] and other groups in FSWs’ immediate environment [138], cannot be described as ‘community-led’, since these efforts remained driven by ‘outsiders’ (project staff). However, attempts at wider community engagement collectively show a promising recognition of the need to address broader structural determinants of FSW vulnerability.

3. *Ownership: By the community*

Our review identified very few examples of true community ownership of FSW SRH interventions. One in particular stands out: the sex worker project in Pumwani Majengo, Nairobi, began with an initial period of engagement and training of peer educators. Subsequently, the communication and representation role of peer-leaders in Nairobi became more formalised, as regular meetings were established with research staff to discuss issues of concern, develop a mutual understanding of community needs, undertake problem solving and develop communication strategies. This in turn legitimised the authority of peer leaders and eventually led to peer-leader elections and establishment of regular democratic forums to allow cohort members to express their concerns and share experiences [68,126-130]. It was these structures that allowed FSWs to self-manage outreach activities, take collective action to tackle internally-identified social problems (for example by requesting microfinance assistance) [126], and build a sense of solidarity and a united front to demand consistent condom use [130]. Project evaluations demonstrated both increased levels of consistent condom use with male clients and partners, as well as reduced incidence of STIs and HIV [34]. Again, it is thought that providing a physical space to create formal democratic structures was vital to this success [130].

4. *Sustaining: By the community*

It is very difficult to comment on the sustainability of SRH and empowerment interventions for FSW in Africa, since longevity data were generally unavailable. Of particular interest is whether peer education projects that achieved high levels of participation and engagement were able to maintain this in the long term. In addition to the likelihood that some project timeframes were limited by research schedules or funding, our review found that retention of peer workers varied greatly across projects, depending on the quality and depth of training and support available. For example, in Mombasa, Kenya, sixty-two FSWs were trained as peer educators over five days in 2000, followed by a six-day advanced course and three-day refresher training. A full-time field coordinator maintained regular contact with peer workers and attended peer education sessions. Five years later over 90% of those originally trained were still active peer workers [145]. By comparison, another study in Malawi reported much lower retention of peer workers. This project covered all districts of the country and was led by district health management teams that struggled with limited resources and ever-increasing demands on their time. Consequently, little support was offered to their cohort of peer workers. Over time, activities in several districts had ceased [137].

Similarly, it is unclear whether early attempts at non-FSW-led wider community engagement were successful in garnering broader support from civil society, which may serve to increase project sustainability. We did find some evidence of acceptance and protection from the surrounding community where interaction between community members and FSWs was encouraged: for example in Malawi where sensitisation sessions with entertainment venue personnel led to their active involvement in promoting safer sex to clientele [138], as well as those projects that recruited a wider range of stakeholders in the sex work industry as peer educators. Nonetheless, according to Ashodaya’s framework of community empowerment, this fourth stage involves, at a minimum, establishment of a community-based organisation with associated formal democratic structures, such as a self-regulatory board, savings schemes, safety mechanisms and broader civic involvement to tackle FSW marginality – none of which were documented in the African projects we reviewed.

## Discussion

Of the SRH interventions assessed in this review, very few appear to have focused on community-led service provision or were concerned with building the capacity of the FSW community to change their circumstances. Our results support findings of a recent review of sex worker community empowerment intervention evaluations in low and middle-income countries by Kerrigan *et al.*[154], which identified no projects in sub-Saharan Africa initiating community empowerment in the sense defined by Wallerstein, namely, as *“a social action process that promotes the participation of people, organizations, and communities towards the goals of increased individual and community control, political efficacy, improved quality of community life, and social justice”*[22].

Although the Ashodaya framework represents only one of many possible evaluation methodologies, it is telling that the vast majority of interventions reviewed had conducted activities to enable empowerment processes consistent with only stage one or two in this framework. Because achievement of each stage was conceptualised as being necessary but not sufficient to allow progression to the next, we did not consider whether individual processes in one stage facilitated achievement of others without following the defined stepwise continuum. An example of where this may have been relevant is the Zambian Corridors of Hope project, within which project staff initiated broader social engagement from the outset (a stage four component that within the Ashodaya framework would be undertaken by FSWs themselves). Similarly, we are aware of some younger African SRH projects that do not engage with FSWs from within the service delivery model (with its inherent patient-provider power relations), but nonetheless aim to impact health service provision by first supporting communities to analyse and act on health and human rights issues concerning them. One example (a study of which did not meet our review criteria) is a UNFPA-supported initiative in Namibia that in 2011 trained sex workers to conduct rapid assessments on HIV and sex work [155].

Notwithstanding apparent limitations of approaches taken by the majority of projects in this review, efforts to build close links with peer networks and involve FSWs in project design and implementation do improve acceptability and participation, a crucial first step in addressing conditions that heighten FSW vulnerability [16]. The significance of these efforts should therefore not be overlooked. Furthermore, the fact that isolated examples of empowerment ‘successes’ exist (i.e. interventions meeting stage two or three) demonstrates proof of concept for the progression of FSW community empowerment in African settings.

One explanation worth considering for why African projects have not (yet) progressed through Ashodaya’s four stages is that these projects have simply not had time to develop complex empowerment processes and the formal structures required to cultivate them. The level of collective organisation required to bring about significant social and political change is usually only achieved after several years of sustained community mobilisation [20]. Indeed the Sonagachi project evolved from a single STI clinic over more than a decade, in response to sex workers expressed concerns [13]. Yet public health and service engagement with African FSW communities has been happening since at least the mid-1990s and has not evolved in the same way as in India [52,156-158].

The fact that the success or failure of certain peer education projects in Africa could be attributed to the level of training and support offered, highlights not only the challenge of resource availability, but also of securing long-term commitment of programmers who facilitate empowerment by engaging and adapting to the needs and capacity of these communities. In most cases we were unable to determine project duration, however the fact that most projects were established as part of research studies, potentially with limited funding and timeframe, is worrisome, as this limits opportunities to develop effective and lasting community empowerment processes. Power differentials between researchers and study participants may also undermine such processes, with researchers primarily viewed as being scientific experts, rather than as being skilled to engage with stakeholders, such as police [20]. Further, although ongoing community engagement is clearly a crucial component of sustainability, our assessment does not consider financial sustainability, which may be of particular concern in Africa due to lack of government involvement and project isolation. In Ashodaya’s case however, successful community engagement itself contributed to financial sustainability, through establishment of an independent cooperative society to provide loans to sex workers and non-sex workers alike, the interest from which was reinvested into Ashodaya.

The socio-political context in which empowerment interventions are implemented is also likely to be a key determinant of their success; differences in the characteristics and organisation of sex work and the overall socio-political context in Africa, compared to India, may therefore limit the effectiveness of activities aiming to mobilise communities, as well as the impact of community mobilisation itself. Cornish and Campbell [159] provide a useful conceptual framework of ‘interventions-in-context’, based on an ecological perspective of community psychology, to examine inconsistent outcomes in peer-education programmes between different settings [159]. Whilst FSWs worldwide have historically been marginalised and disenfranchised, it is arguable that the social fabric, infrastructural, economic and political context in many African settings is exceptionally disempowering [159]. In contrast to India’s relatively structured sex work industry, FSWs across Africa are a highly heterogeneous and mobile group of women with varying frequency, duration and location of sex work, many of whom do not even self-identify as sex workers [3,160]. In addition to this, nascent democracies, criminalisation and weak welfare states leave many in this environment with few foundations from which to unite or assert their rights, indicating that even longer time frames and higher levels of support may be required to facilitate community collectivisation [155,159]. Even for one of Africa’s ‘empowerment successes’, the Pumwani Majengo project in Nairobi, lack of political will and financial resources were initially major constraints to expanding programme coverage, despite strong evidence that this service delivery model could make an important contribution to HIV and STI prevention [68]. To some extent these constraints were, however, subsequently overcome and the project has now been replicated in several Kenyan cities. In terms of community engagement, Pumwani Majengo demonstrates that careful programming can mitigate adverse social contexts. Here community spirit was nurtured by using the STI clinic to promote awareness of the fact that individuals in an outwardly heterogeneous community still shared needs and interests [20,130].

Examination of various SRH service delivery models targeting FSWs provides valuable insights into the way in which sex worker vulnerability is framed on the continent. Ashodaya’s approach encouraged and supported sex workers to re-conceptualise their vocation and demand services to counteract various occupational hazards, thus entrenching bottom-up community empowerment. A range of social interventions, including crisis response and political advocacy, as well as clinic-based SRH promotion were thus provided from the outset [15]. The rarity of structural interventions in African projects suggests a predominant bio-behavioural approach to effecting disease control, rather than a community development approach to service delivery. FSW vulnerability in African settings thus has, to date, been regarded not as an occupational health problem with structural antecedents, but a matter of individual risk behaviour. Arguably a consequence of this framing is that peer education has tended to be treated as a participatory end-point, rather than a means of achieving progressive involvement. By contrast, Sonagachi peer education provided the experiential context that promoted and led to wider experiences of effective community action [161]. ‘Exit programmes’ – which seemingly are rare in Africa – also exemplify activities which may be undertaken in good faith, but whose capacity to empower is limited when the emphasis is on ‘rescue’ from sex work, rather than promoting individual agency [162]. In this respect it is the agenda and purpose of the process, rather than merely the process itself, which determines it’s potential to be simply participatory or truly empowering [20]. Progression through Ashodaya’s framework is therefore not simply an inevitable outcome of certain participatory activities, but may in some cases mandate re-evaluation of a project’s philosophy and a strong commitment to pursuing the social and political changes required to meet the expressed needs of the community.

International public health discourse around SRH in the past decade has moved from a focus on adverse outcomes of sexual behaviour, such as teenage pregnancy and STI transmission, to one of broader SRH and rights [163]. A failure to recognise the socially transformative value of community empowerment processes – independently of their impact on behavioural and clinical outcomes – may lead to effective interventions being overlooked and underfunded [163]. This has implications for study design, since traditional clinical trials are seldom able to account for the complex associations caused by structural interventions [164]. Evaluation frameworks for health interventions are typically short-term in orientation, and therefore unable to accommodate, or even recognise, the longer timeframes needed for community empowerment processes to evolve.

Evidence suggests that no single approach can be used when designing SRH interventions; rather, a multifaceted approach that addresses individual behaviour as well as the social and political context is required [163]. The fact that processes rather than specific activities are prioritised within the Ashodaya framework means that its application is highly context specific and must be tailored to the specific needs of a community [165]. A range of approaches to alter the structural causes of FSW vulnerability has been trialled internationally, in order to identify locally appropriate and effective solutions. For example, Thailand’s 100% Condom Use Programme, which provides outreach to sex workers and mandates condom use in all sex establishments, has increased access to STI services and enabled sex workers to demand condom use. The programme was scaled up nationally in the early 1990s and has achieved marked public health benefits. Various 100% Condom Use Programmes have been successfully implemented in several Asian countries alongside multi-stakeholder mobilisation, to create enabling environments for outreach and increased access to services [165]. In the Dominican Republic, the Sonagachi ‘grassroots’ approach was most effective only in combination with top-down condom legislation akin to 100% Condom Use Programmes [166].

This systematic review has several limitations. Most obviously, we examined only the structure of interventions, rather than uptake or effectiveness. This approach was adopted partly to focus our efforts within a wide subject area, and partly because of the conceptual decision to define empowerment in terms of processes rather than a simplified dichotomy of whether empowerment had been achieved or not. We do acknowledge, however, that alternative theoretical frameworks may be appropriate for further research, particularly for individual project evaluations. Some of our findings allude to potentially important empowerment ‘outcomes’ for assessment: for example, we note that in some circumstances, too wide a forum may have discouraged the most vulnerable from voicing or acting on their concerns, thereby limiting the impact of the intervention. Here, the outcome in question would be the extent to which a range of individuals was able to participate. Other outcomes such as coverage, rates of consistent condom use, or a measure of progressive involvement of FSWs are further examples of important clinical, process and empowerment ‘outcomes’ not examined in our approach.

A second limitation is that by undertaking a systematic review focusing largely on peer-reviewed literature, we have likely excluded a large number of projects in Africa led by non-governmental or community-based organisations, which potentially have closer working relations with FSW communities than researcher- or government-led services. For example, through other research in South Africa, the authors are familiar with the work of SWEAT, and therefore recognise that SWEAT’s activities as reported in the academic literature (and which are detailed in this article) do not do justice to the broad range of activities and complex democratic structures that characterise this organisation in practice [167]. Importantly, SWEAT’s partnership with the sex worker organisation Sisonke has facilitated a national network of sex workers, thus substantially increasing sex worker involvement in project activities. Similarly, the Sex Workers’ Outreach Programme (SWOP) in Kenya runs a nationwide network of drop-in centres, yet was not identified in our literature review [168]. Although not necessarily directly related to SRH service provision, numerous other sex worker associations, such as those affiliated with the African Sex Worker Alliance (ASWA), are also involved in important community development work [169,170]. These activities are known to us because of our familiarity with these organisations, and not because they are described in the published literature.

Notwithstanding these limitations to our search methods, we regard a systematic review of the peer-reviewed literature on this subject to be a valid methodology. A database search of this literature provides a systematic way of surveying evidence on this subject, a difficult task with grey literature. Also, it highlights the need for more research in this field to be published (whether descriptive or evaluative), in order to facilitate informed and transparent project design in the future. The false dichotomy between academic research and community activism should not prevent information sharing that may promote more effective realisation of health and human rights of vulnerable populations.

A third limitation is that we assessed empowerment activities only within the parameters of SRH service provision. As previously stated, adopting wider parameters was considered beyond the scope of this research. However, we caution the reader to be aware that highly valuable efforts to shift social vulnerabilities outside the health sector are potentially excluded here. The fourth limitation concerns the focus on FSWs, with the review unable to assess services targeting the sizable population involved in transactional sex in Africa. Moreover, by focusing on FSW services in Africa as a whole, we have not accounted for the high level of heterogeneity in socio-political contexts between and within countries, thereby limiting the applicability of our conclusions. Finally, some methodological constrains warrant mention. Single data extraction was done, which incurs more errors than duplicate independent extractions. Also, though the review adhered to the principles underlying systematic reviews, it differs from most quantitative systematic reviews, for example in omitting an assessment of risk of bias.

## Conclusions

Although there are clearly lessons to be learnt from previous experiences, there is currently insufficient evidence within the academic literature to support the scale up of any particular model of community empowerment within SRH interventions in African settings. Further rigorous research in this region is needed, using appropriate methods and follow-up periods to measure intervention outcomes longitudinally. Progression through Ashodaya’s framework may not be possible in all African settings, nor can we determine whether collectivisation alone is necessary or sufficient to achieve significant improvements in health and human rights - highlighting the particular need to investigate context-specific, innovative or combination approaches to reduce structural vulnerability of FSWs in Africa.

Whatever the exact configuration of approaches to FSW empowerment in African settings, realistic goals and timeframes are needed to allow empowerment processes to unfold and take root within long-disenfranchised communities. Early and ongoing consultation and involvement of the FSW community is of vital importance. This helps nurture a sense of ownership by the community and commitment by all stakeholders, regarding both the context-specific problems and solutions that the intervention seeks to address. Conversely, involvement of a wide range of stakeholders should be pursued with caution when determining which structural factors to address in a FSW intervention: defining these factors *a priori* may pre-empt the stages of empowerment, in which FSWs themselves identify relevant structural barriers to tackle.

Our findings suggest that FSW SRH interventions in African settings that seek to engage with the community need to rethink their lack of attention to structural factors and reframe FSW vulnerability as an occupational health problem, to be addressed through collective action built on genuine involvement of FSWs themselves. This means moving away from a narrow disease-control focus and creating an environment which is both receptive and responsive to the expressed needs of the community, thereby promoting the transition from technical to transformative communication that facilitates social and political change [161]. Ultimately, higher levels of human and financial resources are needed to ensure sustainability of long-term interventions that are ‘owned’ by African FSW communities in much more meaningful ways than before.

## Competing interests

The authors declare that thay have no competing interests.

## Authors’ contributions

LM, AD and MFC designed the study tools and extracted data from the studies located. FS and LM wrote the first draft of the paper. RS, SRP and FS developed the conceptual framework for the study and assisted in applying the findings to the selected framework. YL and BV provided technical inputs into the study coordination and analysis. All authors helped draft the final paper and approved the final manuscript.

## Supplementary Material

Additional file 1: Table S1 Community-level interventions and sex worker involvement within targeted SRH interventions for female sex workers in Africa. Click here for file
