# Supplementary material for: Community empowerment and involvement of female sex workers in targeted sexual and reproductive health interventions in Africa: a systematic review
Source: Global Health. 2014 Jun 10;10:47. doi: 10.1186/1744-8603-10-47 (PMC4074148; doi:10.1186/1744-8603-10-47)
Supplement: Additional file 1: Table S1 — Community-level interventions and sex worker involvement within targeted SRH interventions for female sex workers in Africa. [file 1744-8603-10-47-S1.docx]

**Additional file 1: Table S1: Community-level interventions and sex worker involvement within targeted SRH interventions for female sex workers in Africa**

| **Country, City** | **Target group** | **Facility and setting** | **Description of community-level interventions** | **Role of SWs in services/projects** | **Other Human Resources used in services** |
| --- | --- | --- | --- | --- | --- |
| Benin, Cotonou, Porto Novo, 10 small town clinics across country [[1-13](#_ENREF_1)] | FSW and their male partners | A dedicated clinic & visits at home/ work by fieldworkers. One specialized STI SW clinic in Cotonou (opened in 2000), two in Porto Novo and 10 other small town clinics) | Peer education. Outreach/community workers (often ex-SW), with close links with FSWs, recruit women to the clinic for STI diagnosis and treatment, and carry out educational and awareness-raising workshops in the field, including training of peer educators. Projet SIDA-2 trained brothel-owners and some men, recruited from within the SW milieu in Cotonou, for HIV/STI education targeting male clients and regular partners of FSW in bars, hotels, and streets, in areas with a high FSW concentration. (Projet SIDA-3 now underway). | Since 1999, an association of FSW, funded and supervised by Projet Sida-2, has done outreach to increase HIV/STI awareness, empower FSW in condom negotiation with clients and boyfriends, and refer newcomers to STI clinic. | Field workers who approached SW in homes and at work to recruit them; also approached male clients at SW sites |
| Burkina Faso, Bobo-Dioulasso [[9](#_ENREF_9), [14-18](#_ENREF_14)] | Professional (>5 clients/ week) and non-professional FSW | Dedicated clinic within a general public health facility. | Peer educators recruited and trained by study staff to educate on condom use (incl. negotiation skills), HIV and STI infections. | Peer educators doing outreach. | Study staff recruited and trained the peer educators. |
| Cote d' Ivoire, Abidjan, and 11 other towns [[19-29](#_ENREF_19)] | FSW | 2 Confidential FSW clinics in a non-stigmatizing and confidential environment; research centre; and 11 PHC clinics with an integrated package of services for SW | Network of peer educators, promoting and demonstrating condom use, STI and HIV prevention. Outreach activities by clinic personnel. Education activities by peer workers, including picture album tool, video films, drawings for education, condom demonstration and provision, group education in community. In outreach, FSW are invited to clinic for STI assessments. | Peer educators are current and former sex workers. | Clinic personnel |
| Democratic Republic of Congo, Kinshasa, Matonge [[30](#_ENREF_30)] | Not stated | A special women's health centre, FSW and STI clinic | Peer education and outreach activities in the community encouraging SW to visit the clinic | Not stated | Physician performs clinical exam, including pelvic exam |
| East and Central African highways [[31](#_ENREF_31)] | No specific mention of FSW targeting, but provides recreation and resource centres, with educational outreach, HTC and secure place to relax for truck drivers and other transient workers | Transport corridors in Burundi, Democratic Republic of Congo, Djibouti, Ethiopia, Kenya, Rwanda, Sudan, Tanzania and Uganda. | Peer-based family planning, health education, and referrals (not specifically for FSWs). | Not stated | Not stated |
| Ghana, Accra [[32](#_ENREF_32)] | FSW | Community-based outreach intervention | FSWs and non-paying partners trained as peer educators, provide basic health promotion in homes and communities of sex workers. Trained nurse and HIV counsellor provide HCT. | Peer educators | Nurse and HIV counsellor  Non-paying partners trained as peer educators |
| Ghana, Accra, Kumasi, Techiman [[5](#_ENREF_5), [33-38](#_ENREF_33)] | FSW | Dedicated clinics for FSWs, Ghana Red Cross drop-in centres. Initially Accra, extended to clinics in 3 cities | FSWs approached by community health nurses or peer educators who invited them to participate in HIV/STI program. Field workers visited FSWs at homes. Group meetings in SW communities, bars and clinic for continued education sessions conducted by the trainer, physician and outreach staff. | Peer educators | Community health nurses, peer educators. Medical doctor or midwife for pelvic examination |
| Guinea, multiple sites including Conakry [[39](#_ENREF_39), [40](#_ENREF_40)]  *Implemented in collaboration with the West Africa AIDS Program AIDS-3* | FSW and clients | Adapted Health Services (AHS) offer medical care and assistance adapted to the specific needs of FSWs. Integrated into general health services to avoid stigma. | Projet SIDA-3 supports health centres in the development of adapted services. Also supports local community-based NGOs carrying out fieldwork to promote condom use and health seeking behaviours. As part of HCT acceptability study FSWs were contacted at their workplace or at home where HCT undertaken and free condoms distributed. | FSWs are expected to visit an AHS at least once a month in order to have a valid health booklet. | Field workers, clinical staff working at clinics. |
| Kenya, Kisumu [[41](#_ENREF_41)] | Not stated | Integrated clinic, free services. Intervention modelled after clinic in Cote d’Ivoire | Peer-led outreach | Not stated | Not stated |
| Kenya, Mombasa, Kisauni [[42-47](#_ENREF_42)] | FSW | Peer education in FSWs houses or at a drop-in centre within community | Peer educators conducted 1-on-1 or weekly-group sessions, mostly in FSWs’ houses or at a drop-in centre based within community, using peer-mediated drama, role playing exercises, use of picture codes and video sessions to provide HIV education, condom promotion and other risk-reduction activities. Community gatherings with active participation of FSW, youth and other community members. Drop-in centre used for distributing IEC materials and condoms | As peer workers: recruited from FSW in the area, aiming to include FSW who were willing to be a peer leader, had a substantial network of peers, were likely to remain in the area for an extended period, and had some knowledge of the key topics.  Peer educators (n=62) were trained, recruited in 2000, 57 retained until 2005. Training: 5-day course, 6-day advanced and 3-day refresher course midway in project. | A field coordinator updated peer educators and regularly attended peer-education sessions for monitoring. |
| Kenya, Nairobi, Mukuru [[48](#_ENREF_48)] | FSW and male clients | Mukuru community health clinic | Community support gained through meetings with local administrators and village elders. FSW participants invited to attend meetings. Community health education. | FSW invited to presentations about research studies. | Not stated |
| Kenya, Nairobi, Kibera [[49-54](#_ENREF_49)] | SW | Nairobi research clinic (PHC model) | SW community meetings organized quarterly in each of the 10 villages comprising the Kibera slum. 6-monthly wider meetings of SW from all Kibera villages. Peer-mediated education programme. | As peer educators | Not stated |
| Kenya, Nairobi, Pumwani Majengo [[55-60](#_ENREF_55)] | FSW cohort | PHC clinic and community outreach programme. Free comprehensive health care. Expanded to include other slum area and 3 other outreach towns. | Health education at general community meetings for FSW and all women in community every 6 months. These included STI effects and prevention methods through lectures, skits, role-playing and discussion. Community outreach process: 1) entry into community using key informants 2) Public meeting with FSW to create community links and establish leadership and groups, which then meet weekly 3) Train peer educators and counsellors on condom promotion, sexual risk reduction and STI/HIV risk reduction 4) Individual and group counselling. Each peer educator responsible for about 20 peers. Peer-led education includes condom promotion, importance of partner reduction, avoiding unprotected vaginal and anal sex, increasing non-penetrative sex and avoiding sex during menses. Elected peer-leaders with formal space for all peer leaders to periodically meet, solve problems, make communication materials. Microfinance project provided FSWs with credit for small businesses, business skills training and mentorship. | FSW elected as members of committee to oversee study, decide priorities and communicate with researchers. Elected representatives also work as peer educators and counsellors. | Social and health (physicians, nurses, assistants) workers at PHC, peer educators in community. Peer leaders elected by other FSWs to be their representatives. Clinic staff trained in community mobilisation, counselling skills and syndromic STI management. |
| Kenya, trans-Africa highway [[61](#_ENREF_61)] | FSW, truck drivers and their assistants, young men living and working at truck stops | 3 stops along highway, with 1000, 2000, 5000 inhabitants. | Not stated | As participants, recruited via snowballing. | Interview moderator - native Swahili speaker. Focus group moderator - native Kenyan who was respected and well known at each truck stop for his public health work. |
| Madagascar, Antananarivo, Tamatave, Antsiranana, Mahajanga, Toamasina [[62-73](#_ENREF_62)] | FSW | Public dispensary, non-government facility provides community-based education and clinical services to STI patients and FSW (''67 Ha Clinic''). Counselling also in community. | Peer education (community-based outreach). FSWs contacted in community by peer educators and invited for free STI care. Peer educators do condom promotion and risk reduction counselling in clinic and community. Prevention messages and communication strategies developed in consultation with peer educators. Clinic counsellors had 2 week-long trainings that covered basic elements of effective counselling, including understanding of values, identifying and assessing stages of behaviour change, tailoring messages, and developing skills for effective interpersonal communication, as well as implementation of study messages | FSW did outreach, acting as peer educators and advisors. Peer educators accompanied FSWs to clinic visits and were intensively trained in counselling. Peer educators received continuing education, supervision and motivation at monthly meetings with the study coordinator and weekly meetings with clinic staff. Project design based on FSWs' views. FSW representatives participated in a 3-day workshop, presenting their perspectives.  Active engagement of peer educators in the research and in decision-making workshop was positive experience for both peer educators & health professionals. | Researchers, policy makers, clinicians & FSW developed guidelines. Clinic counselling by physician trained by consultant specialising in behaviour change communication. |
| Madagascar, Diego-Suarez [[74](#_ENREF_74), [75](#_ENREF_75)] | FSW | Not stated | Community-based education, using FSW peer educators acting as fieldworkers to promote consistent condom use and STI care seeking among FSW. Some FSW in Diego-Suarez are registered with the association Fikambanan’ny Vehivavy Mpandeha An-Tsambo (FIVMATA), or “Women Who Go to the Boats,” and frequent the port and various nightclubs. Clients of registered SW are generally sailors or tourists. Registered FSWs carry a health card and are required to make regular visits to the public STI clinic to gain access to the port. Since 1987, approx. 1200 SWs have registered with FIVMATA. | Peer educators. FSWs have also been involved in drawing up of guidelines. | Clinicians |
| Malawi, 3 towns in Thylo District (Thyolo, Luchenza and Bvumbe) [[76](#_ENREF_76), [77](#_ENREF_77)] | FSW registered at bars and rest houses as beer-servers/cleaners/food handlers. | Services provided via mobile clinic at designated rest house facility. Room available for medical examinations. | Mobile clinic staffed by 2 STI clinicians and experienced counsellors. Peer outreach workers mobilise FSW to attend clinic | Peer educators recruited to encourage FSW to attend mobile clinic. | District health team runs clinic. Team of 2 trained STI clinicians provided mobile clinic services. |
| Malawi, Dowa & Lilongwe Districts (3 district towns adjacent to capital (Lilongwe) & on main trucking routes from Tanzania & Zambia [[78](#_ENREF_78)] | FSW | Peer services provided in bars. Program expanded nationwide. | Peer education, including info about HIV, condom promotion and distribution, and safe sex negotiations skills for FSW. Health talks given by health staff to FSW groups | Peer educators attended 4-day training. | Health staff |
| Malawi [[79](#_ENREF_79)] | Sex workers in places of entertainment (e.g. disco houses, pubs) | Intervention focused on places of entertainment | Project sought cooperation with bar owners, DJs and SWs. Sensitisation sessions and workshop with SWs, DJs, and club owners, to gain support. Information on HIV shared via presentations, IEC materials, discussions and testimonies. Action groups formed with leaders for training as peer educators. DJs distributed safe sex messages. Weekly condom and safer sex promotional events with quizzes and awards. Sex worker Safer Sex Kit distributed. STI services. | Peer educators had 6 day training on HIV, negotiating safer sex, male & female condom use, sexual health rights and how to fight violence/abuse/exploitation, options for income generation | Not stated |
| Mozambique, Tete, Moatize [[80](#_ENREF_80)] | SW and truck drivers | Clinic in 2 converted shipping containers near major truck stop ("Tete Corridor" connecting Zimbabwe with Malawi) on outskirts of Moatize town, 20km from Tete | Peer educators provide behaviour change communication and condom distribution through outreach. | Peer educators doing outreach, also contributed input into project design, along with clients | ICRH staff (International Centre for Reproductive Health) |
| Mozambique, all provinces [[81](#_ENREF_81), [82](#_ENREF_82)] | Truckers, military personnel, FSW | Condom social marketing | Condom distribution at non-traditional outlets, meeting places for men and women in non-regular partnerships (hotels, bars, restaurants and nightclubs), such as gas stations, kiosks, tobacconists, supermarkets, workplaces, itinerant traders and pushcart salesmen. Peer education debates and theatre performances. | Peer educators | Network of community based agents for behaviour change activities. |
| Namibia, Oshakati [[83](#_ENREF_83)] | ‘High- risk groups' such as female sex workers | Not stated | Peer educators run female-only and co-ed sessions in gender-neutral spaces aiming to empower female sex workers to actively negotiate sexual decision-making using knowledge, attitudes, behaviour approach: question/answer opportunities, condom use demonstrations, role-playing exercises, and attempts to disband myths and stereotypes. | Peer educators trained to provide BCC sessions. | Not stated |
| Nigeria, Cross River State, Calabar and Ikom. Also Port Harcourt, Rivers State [[84](#_ENREF_84)] | FSW | STI clinic mainly, also other health and preventative services | In hotels where FSW stay, monthly ''chairladies meetings'' and regular ''proprietors meetings'' held to gain involvement in project implementation and serve as a forum to discuss issues. Health education sessions in hotels. Condoms distributed at sites. | FSW elect chairlady for each hotel and are consulted and give input into project in general. FSW are also trained peer educators. | Focus on ''Gatekeeper'' individuals who live in SW environment, provide SW with services, advice or protection, and hold some influence. May include local security agents and police commissioner. Health workers involved in project and STI clinic (nurse and health educator). |
| Nigeria, Jos [[85](#_ENREF_85)] | Brothel-based FSWs living in urban Jos-Bukuru environs. | Project involved a private women's hospital in Jos | Not stated | Representatives of FSW community consulted about study design and setting. | Gynaecologist performing examinations. |
| Rwanda, Kigali [[86-89](#_ENREF_86)] | FSW | Research NGO, with research clinic and laboratory | Not stated | Some FSW worked as community mobilisers (prominent community members with extensive social networks), leading project meetings. | Outreach staff assisted by leaders of local associations for FSW and community mobilisers. |
| Senegal, Dakar [[90-92](#_ENREF_90)] | FSW | FSW required to register with 1 of 4 national specialist medical centres for FSW. Services began after National AIDS Prevention Committee in 1986 to respond to HIV epidemic. | Projet SIDA-3 supports health centres in the development of adapted services. Also supports local community-based NGOs carrying out fieldwork to promote condom use and health seeking behaviours. | Peer educators sometimes run safe sex classes at the centre. FSW register at clinic for monthly medical follow up and exam. Safe-sex classes run by Association for Women at risk from AIDS. | Doctors, nurses, social workers, receptionist |
| South Africa, Johannesburg, Hillbrow [[12](#_ENREF_12), [93-97](#_ENREF_93)] | SW | Mobile and permanent clinic | Mobile outreach provides range of clinical services. IEC activities. Community health workers assist clients, give health talks to clients. Exit programme where peer educators have skills training in alternative employment | Peer educators | Trained PHC nurse and counsellors, community health workers |
| South Africa, Cape Town [[98](#_ENREF_98)]  *Sex Worker Education and Advocacy Taskforce, SWEAT* | SW | Programme based at NGO offices | NGO developed outreach programme for street-based FSW. FSW work as volunteers in outreach programme. PHC/STI clinic staff members join outreach visits. NGO built a working relationship with clinics, provided training to staff, who then accompanied fieldworkers on outreach visits. NGO and community lobbied for more public cleaning and toilet facilities. Initiated intervention with gatekeepers (pimps or gangs), so they support and accompany FSWs to drop-in centre. Gatekeepers/pimps included in health education programs. Pimps given educational material and condoms, protect outreach health educators on late night shift. | 2 FSW and 2 former FSW involved in fieldwork. FSW outreach workers (volunteers) educated about legal issues. | Non FSW fieldworkers, clinic staff |
| South Africa, Carletonville [[99](#_ENREF_99)] | Mine workers and FSW | Two fully staffed and equipped mobile clinics made monthly visits to major sites where mine workers meet FSW | Community-level intervention to control HIV and STI spread, involving peer educators from SW, mine workers & youth. | Peer educators from among local FSW (as well as mine workers and youth) recruited and trained. | Not stated |
| South Africa, Durban-Johannesburg highway, truck stops [[12](#_ENREF_12), [100-108](#_ENREF_100)] | FSW | 5 truck stops on road between Johannesburg and Durban | Not stated | Elected FSW employed in project | Not stated |
| South Africa, Free State, Virginia town, Harmony Mine [[109-111](#_ENREF_109)]  *Lesedi Project* | FSW, mine workers and high- risk women | Mobile clinic served township and fixed clinic based in mine-owned house. Also community outreach and health promotion. Multi-sector approach, project grew over time, partnership with mining companies. | Clinic based STI testing and diagnosis plus periodic presumptive treatment of STIs for those at high risk. Formative research helped to tailor services to be more acceptable to FSWs.  Outreach workers approached FSW through proprietors of meeting places and distributed clinic referral cards.  6 peer educators trained to provide information about sexual risk reduction, condom use and advantages of clinical services. Peers also distributed clinic referral cards to FSW, encouraged them to attend mobile clinic monthly. In 1-on-1 and group meetings, peers explained services available at clinic and built community trust and involvement in program.  The intervention is now being replicated in other  mining regions in South Africa (including Carltonville) | Peer educators: participated in project design and met weekly with the nurse to plan activities and discuss solutions to problems encountered. | Professional nurses provided STI services. Also project outreach workers, peer education trainer. Medical director. |
| South Africa, Mpumalanga [[112](#_ENREF_112)] | Occasional and full-time commercial FSW, as well as women engaged in multiple casual, but non-commercial, partnerships. | Outreach projects with peer educators in 35 projects within the province. | Project aims to promote HIV awareness and behavioural change through a peer process of condom promotion, STD prevention and HIV risk awareness. Outreach focuses on women who supplement limited agricultural income with occasional commercial sex, whereas other projects are geared for women for whom commercial sex is a full-time occupation. | Peer workers | Not stated |
| South Africa, Pretoria [[113](#_ENREF_113), [114](#_ENREF_114)] | Not stated | Not stated | CAB meetings | Not stated | Not stated |
| Southern Africa, Durban-Lusaka highway, Northern Province highways [[115](#_ENREF_115)] | High-risk populations at borders and highways | Peer education programme | Peer education programme and other services managed by interdisciplinary district committees | Not stated | Not stated |
| Tanzania, highway [[116](#_ENREF_116)] | 330 high-risk women, truck drivers and 3 trucking companies in Dar es Salaam | 7 truck stops on highway from Dar es Salaam to Zambia | These truck stops were already participating in an existing HIV prevention programme. Local health care provider provided special STD services twice a week outside normal hours. Team of clinicians provided outreach STD services 3mnthly at a site away from health facility. Peer health educators at truck stops engaged with other FSW and truck drivers, providing health education, condom promotion and referral cards to clinics if needed. | Peer educators were mostly FSW. At the truck stops, peer educators were female bar and guesthouse workers and male petrol station workers. At trucking companies peer educators were usually social welfare and transport officers. | Clinicians at PHC facility providing STD care, local health care provider. Peer educators were identified, trained and coached by two Master s degree level health behaviour officers. |
| Togo, Lome and other urban centres [[117](#_ENREF_117)] | FSW | A clinic in the capital city of Lome, other towns | Peer educators provided outreach in distributing condoms, recruiting FSW at sites, and mapping SW sites | FSW as peer educators conducted mapping of sites, enumeration of SWs, recruitment of SW. | Male field workers visited SW sites to recruit clients |
| Uganda, Kampala, Kibuye (densely populated slum) [[118](#_ENREF_118)] | Not stated | Stand-alone clinic offering free general and reproductive health care. On site laboratory. | Not stated | Study group collaborated with local SW NGO to recruit peer educators who receive monthly allowance | Field workers visited SW in their workplace to confirm SW identity |
| West Africa, highways (Cote D’Ivoire, Ghana, Nigeria, Benin, Togo)  [[119](#_ENREF_119)] | FSW | Abidjan-Lagos transport corridor across five countries, with major truck stops and borders | Community-based HIV/AIDS support and care. Members of NGOs trained as psychological advisers. Establishment of border committees and training them for peer-to-peer learning - border communities were mobilized and trained for community mobilization and awareness. MF: trained peer educators | FSW manned 625 sales points for condoms, including 8 border posts. FSWs at sales points trained in condom distribution. FSWs also served as peer educators and attended training sessions. | 5 heads of state endorsed project. Transport authorities counselled. Governing body, advisory body, and executive secretariat monitored overall project implementation. Community-based border HIV committees, inter-country facilitation committees. Civil society and public sector representatives from all countries. Monitoring and Evaluation specialists. Consulting firms to monitor progress of HIV program and transport component. Bank managers. Inter-border Facilitation Committees; all managed by border officials. |
| West Africa: Benin, Burkina Faso, Ghana, Mali, Niger, Senegal, Togo [[120](#_ENREF_120), [121](#_ENREF_121)]  *West Africa Project to Combat AIDS and STIs (AIDS-3)* | FSW and other high- risk groups | Not stated | Community participation to encourage activities promoting health and local responses to HIV. Support organizations that offer alternatives and contribute to creating coalitions that promote health. National programs designed as an integrated approach, combining varying degrees of adapted services and community support targeting SW environments. | Peer educators | Not stated |
| Zambia, Livingstone, border towns and corridor communities [[122](#_ENREF_122)]  *Corridors of Hope Programme* | FSW | Clinics and mobile units. Mobile units provide STI and HIV outreach services such as HCT. Facility centrally located in a nondescript house. | Peer educator program disseminated messages among target groups and the broader community. Project staff came to talk to FSWs in their guest house weekly, sometimes more often. Group sessions employed a reflective, participatory methodology to change individual risk behaviour. Sessions sought to help SW meaningfully participate in decisions about their own lives by helping them reflect on and create action plans to address their own concerns. Program used participatory tools to create open, democratic environment for sharing and to stimulate discussion. Emphasis on dialogue and action, raising awareness, cooperation, and empowerment, working with community members to reduce stigma and discrimination against sex workers. To bring about social change, a social mobilization approach based on community participation and action was used. Community groups worked collaboratively to spread prevention messages among their members to change broader social norms around safer sex practices. | Not stated | Worked with “queen mothers,” women in their 30-40s, often former sex workers, who landlords hired to supervise SWs in guest houses.  Each project site had a Behaviour Change Communication Steering Committee of local organizations to ensure that community members, particularly vulnerable groups, were represented in program activities |
| Zimbabwe, Bulawayo [[55](#_ENREF_55), [123](#_ENREF_123)] | FSW and male clients | Not stated | Peer group educators chosen to educate and distribute condoms among SWs. Trained in STIs and HIV IEC techniques, condom promotion and community mobilization.  Peer educators divided into 7 zones, reflecting geographic and demographic makeup of Bulawayo. Each zone functions as a cohesive unit and has a senior peer educator or group leader responsible for organizing, motivating, and supervising peer educators. | Not stated | Bar security and sales personnel used as health educators. Also, male peer educators recruited and trained from vulnerable groups of men, including clients of FSWs. |
| Zimbabwe, Shurugwi, Midlands mining town [[124](#_ENREF_124)] | FSWs in Shurugwi (rural town with mining compounds). | Services at 2 hospitals, referral sites of mining clinics | Researcher gave 'lectures' on STDs and their complications in beerhalls, community halls, work places and commercial farms | Not stated | SW examined by medical officer and by psychiatric nurse for HIV T&C. Public health officer from mining company. Government health inspector and researchers. |

Abbreviations: FSW – female sex worker; HTC – HIV testing and counselling; IEC – information, education and communication; NGO – non-governmental organisation; STI – sexually transmitted infections; SW – sex worker; PHC – primary health care.

**References**

1. Mukenge-Tshibaka L, Alary M, Bernier F, van Dyck E, Lowndes CM, Guedou A, Anagonou S, Joly JR: **Diagnostic performance of the Roche AMPLICOR PCR in detecting Neisseria gonorrhoeae in genitourinary specimens from female sex workers in Cotonou, Benin.** *J Clin Microbiol* 2000, **38:**4076-4079.

2. Mukenge-Tshibaka L, Alary M, Lowndes CM, Van Dyck E, Guedou A, Geraldo N, Anagonou S, Lafia E, Joly JR: **Syndromic versus laboratory-based diagnosis of cervical infections among female sex workers in Benin: implications of nonattendance for return visits.** *Sex Transm Dis* 2002, **29:**324-330.

3. Alary M, Lowndes CM, Mukenge-Tshibaka L, Gnintoungbe CA, Bedard E, Geraldo N, Jossou P, Lafia E, Bernier F, Baganizi E, et al: **Sexually transmitted infections in male clients of female sex workers in Benin: risk factors and reassessment of the leucocyte esterase dipstick for screening of urethral infections.** *Sex Transm Infect* 2003, **79:**388-392.

4. Alary M, Mukenge-Tshibaka L, Bernier F, Geraldo N, Lowndes CM, Meda H, Gnintoungbe CA, Anagonou S, Joly JR: **Decline in the prevalence of HIV and sexually transmitted diseases among female sex workers in Cotonou, Benin, 1993-1999.** *AIDS* 2002, **16:**463-470.

5. Pepin J, Labbe AC, Khonde N, Deslandes S, Alary M, Dzokoto A, Asamoah-Adu C, Meda H, Frost E: **Mycoplasma genitalium: an organism commonly associated with cervicitis among west African sex workers.** *Sex Transm Infect* 2005, **81:**67-72.

6. Ahoyo AB, Alary M, Meda H, Ndour M, Batona G, Bitera R, Adjoni C, Medegan VK, Labbe AC, Adjimon T: **[Female sex workers in Benin, 2002. Behavioural survey and HIV and other STI screening].** *Sante* 2007, **17:**143-151.

7. Lowndes CM, Alary M, Gnintoungbe CA, Bedard E, Mukenge L, Geraldo N, Jossou P, Lafia E, Bernier F, Baganizi E, et al: **Management of sexually transmitted diseases and HIV prevention in men at high risk: targeting clients and non-paying sexual partners of female sex workers in Benin.** *AIDS* 2000, **14:**2523-2534.

8. Lowndes CM, Alary M, Labbe AC, Gnintoungbe C, Belleau M, Mukenge L, Meda H, Ndour M, Anagonou S, Gbaguidi A: **Interventions among male clients of female sex workers in Benin, West Africa: an essential component of targeted HIV preventive interventions.** *Sex Transm Infect* 2007, **83:**577-581.

9. Nagot N, Ouedraogo A, Ouangre A, Cartoux M, Defer MC, Meda N, Van de Perre P: **Is sexually transmitted infection management among sex workers still able to mitigate the spread of HIV infection in West Africa?** *J Acquir Immune Defic Syndr* 2005, **39:**454-458.

10. Germain M, Alary M, Guedeme A, Padonou F, Davo N, Adjovi C, Van Dyck E, Joly JR, Mahony JB: **Evaluation of a screening algorithm for the diagnosis of genital infections with Neisseria gonorrhoeae and Chlamydia trachomatis among female sexworkers in Benin.** *Sex Transm Dis* 1997, **24:**109-115.

11. Lowndes CM, Alary M, Meda H, Gnintoungbe CA, Mukenge-Tshibaka L, Adjovi C, Buve A, Morison L, Laourou M, Kanhonou L, Anagonou S: **Role of core and bridging groups in the transmission dynamics of HIV and STIs in Cotonou, Benin, West Africa.** *Sex Transm Infect* 2002, **78 Suppl 1:**i69-77.

12. Van Damme L, Ramjee G, Alary M, Vuylsteke B, Chandeying V, Rees H, Sirivongrangson P, Mukenge-Tshibaka L, Ettiegne-Traore V, Uaheowitchai C, et al: **Effectiveness of COL-1492, a nonoxynol-9 vaginal gel, on HIV-1 transmission in female sex workers: a randomised controlled trial.** *Lancet* 2002, **360:**971-977.

13. Labbe AC, Pepin J, Khonde N, Dzokoto A, Meda H, Asamoah-Adu C, Mayaud P, Mabey D, Demers E, Alary M: **Periodical antibiotic treatment for the control of gonococcal and chlamydial infections among sex workers in Benin and Ghana: a cluster-randomized placebo-controlled trial.** *Sex Transm Dis* 2012, **39:**253-259.

14. Huet C, Ouedraogo A, Konate I, Traore I, Rouet F, Kabore A, Sanon A, Mayaud P, Van de Perre P, Nagot N: **Long-term virological, immunological and mortality outcomes in a cohort of HIV-infected female sex workers treated with highly active antiretroviral therapy in Africa.** *BMC public health* 2011, **11:**700.

15. Nagot N, Ouangre A, Ouedraogo A, Cartoux M, Huygens P, Defer MC, Zekiba T, Meda N, Van de Perre P: **Spectrum of commercial sex activity in Burkina Faso: classification model and risk of exposure to HIV.** *J Acquir Immune Defic Syndr* 2002, **29:**517-521.

16. Nagot N, Ouedraogo A, Defer MC, Vallo R, Mayaud P, Van de Perre P: **Association between bacterial vaginosis and Herpes simplex virus type-2 infection: implications for HIV acquisition studies.** *Sex Transm Infect* 2007, **83:**365-368.

17. Damay A, Didelot-Rousseau MN, Costes V, Konate I, Ouedraogo A, Nagot N, Foulongne V, Van de Perre P, Mayaud P, Segondy M: **Viral load and physical status of human papillomavirus (HPV) 18 in cervical samples from female sex workers infected with HPV 18 in Burkina Faso.** *J Med Virol* 2009, **81:**1786-1791.

18. Konate I, Traore L, Ouedraogo A, Sanon A, Diallo R, Ouedraogo JL, Huet C, Millogo I, Andonaba JB, Mayaud P, et al: **Linking HIV prevention and care for community interventions among high-risk women in Burkina Faso--the ARNS 1222 "Yerelon" cohort.** *J Acquir Immune Defic Syndr* 2011, **57 Suppl 1:**S50-54.

19. Ghys PD, Diallo MO, Ettiegne-Traore V, Satten GA, Anoma CK, Maurice C, Kadjo JC, Coulibaly IM, Wiktor SZ, Greenberg AE, Laga M: **Effect of interventions to control sexually transmitted disease on the incidence of HIV infection in female sex workers.** *AIDS* 2001, **15:**1421-1431.

20. Ghys PD, Jenkins C, Pisani E: **HIV surveillance among female sex workers.** *AIDS* 2001, **15 Suppl 3:**S33-40.

21. Diallo MO, Ghys PD, Vuylsteke B, Ettiegne-Traore V, Gnaore E, Soroh D, Kadjo JC, Van Dyck E, De Cock KM, Greenberg AE, Laga M: **Evaluation of simple diagnostic algorithms for Neisseria gonorrhoeae and Chlamydia trachomatis cervical infections in female sex workers in Abidjan, Cote d'Ivoire.** *Sex Transm Infect* 1998, **74 Suppl 1:**S106-111.

22. Vuylsteke B, Ghys PD, Mah-bi G, Konan Y, Traore M, Wiktor SZ, Laga M: **Where do sex workers go for health care? A community based study in Abidjan, Cote d'Ivoire.** *Sex Transm Infect* 2001, **77:**351-352.

23. Vuylsteke B, Semde G, Sika L, Crucitti T, Ettiegne Traore V, Buve A, Laga M: **HIV and STI prevalence among female sex workers in Cote d'Ivoire: why targeted prevention programs should be continued and strengthened.** *PloS one* 2012, **7:**e32627.

24. Vuylsteke B, Traore M, Mah-Bi G, Konan Y, Ghys P, Diarra J, Laga M: **Quality of sexually transmitted infections services for female sex workers in Abidjan, Cote d'Ivoire.** *Trop Med Int Health* 2004, **9:**638-643.

25. Vuylsteke BL, Ettiegne-Traore V, Anoma CK, Bandama C, Ghys PD, Maurice CE, Van Dyck E, Wiktor SZ, Laga M: **Assessment of the validity of and adherence to sexually transmitted infection algorithms at a female sex worker clinic in Abidjan, Cote d'Ivoire.** *Sex Transm Dis* 2003, **30:**284-291.

26. Vuylsteke BL, Ghys PD, Traore M, Konan Y, Mah-Bi G, Maurice C, Soroh D, Diarra JN, Roels TH, Laga M: **HIV prevalence and risk behavior among clients of female sex workers in Abidjan, Cote d'Ivoire.** *AIDS* 2003, **17:**1691-1694.

27. Ettiegne-Traore V, Ghys PD, Maurice C, Hoyi-Adonsou YM, Soroh D, Adom ML, Teurquetil MJ, Diallo MO, Laga M, Greenberg AE: **Evaluation of an HIV saliva test for the detection of HIV-1 and HIV-2 antibodies in high-risk populations in Abidjan, Cote d'Ivoire.** *Int J STD AIDS* 1998, **9:**173-174.

28. Ghys PD, Fransen K, Diallo MO, Ettiegne-Traore V, Coulibaly IM, Yeboue KM, Kalish ML, Maurice C, Whitaker JP, Greenberg AE, Laga M: **The associations between cervicovaginal HIV shedding, sexually transmitted diseases and immunosuppression in female sex workers in Abidjan, Cote d'Ivoire.** *AIDS* 1997, **11:**F85-93.

29. Mastro TD: **Increase in condom use and decline in HIV and sexually transmitted diseases among female sex workers in Abidjan, Cote d'Ivoire, 1991-1998, by Ghys et al.** *AIDS* 2003, **17 Suppl 4:**S121-122.

30. Mann JM, Nzilambi N, Piot P, Bosenge N, Kalala M, Francis H, Colebunders RC, Azila PK, Curran JW, Quinn TC: **HIV infection and associated risk factors in female prostitutes in Kinshasa, Zaire.** *Aids* 1988, **2:**249-254.

31. Morris CN, Morris SR, Ferguson AG: **Sexual behavior of female sex workers and access to condoms in Kenya and Uganda on the Trans-Africa highway.** *AIDS Behav* 2009, **13:**860-865.

32. Papworth V: **Screening hits the streets.** *Nurs Stand* 2009, **24:**24-25.

33. Cote AM, Sobela F, Dzokoto A, Nzambi K, Asamoah-Adu C, Labbe AC, Masse B, Mensah J, Frost E, Pepin J: **Transactional sex is the driving force in the dynamics of HIV in Accra, Ghana.** *AIDS* 2004, **18:**917-925.

34. Adu-Oppong A, Grimes RM, Ross MW, Risser J, Kessie G: **Social and behavioral determinants of consistent condom use among female commercial sex workers in Ghana.** *AIDS Educ Prev* 2007, **19:**160-172.

35. Asamoah-Adu A, Weir S, Pappoe M, Kanlisi N, Neequaye A, Lamptey P: **Evaluation of a targeted AIDS prevention intervention to increase condom use among prostitutes in Ghana.** *Aids* 1994, **8:**239-246.

36. Asamoah-Adu C, Khonde N, Avorkliah M, Bekoe V, Alary M, Mondor M, Frost E, Deceuninck G, Asamoah-Adu A, Pepin J: **HIV infection among sex workers in Accra: need to target new recruits entering the trade.** *J Acquir Immune Defic Syndr* 2001, **28:**358-366.

37. Deceuninck G, Asamoah-Adu C, Khonde N, Pepin J, Frost EH, Deslandes S, Asamoah-Adu A, Bekoe V, Alary M: **Improvement of clinical algorithms for the diagnosis of Neisseria gonorrhoeae and Chlamydia trachomatis by the use of Gram-stained smears among female sex workers in Accra, Ghana.** *Sex Transm Dis* 2000, **27:**401-410.

38. Akumatey B, MacQueen KM, Guest G: **Condom use and HIV prevention among female sex workers in Tema, Ghana. Abstract no. C11518.** In *Book Condom use and HIV prevention among female sex workers in Tema, Ghana. Abstract no. C11518* (Editor ed.^eds.). City; 2004.

39. Godin G, Tinka Bah A, Sow A, Minani I, Morin D, Alary M: **Correlates of condom use among sex workers and their boyfriends in three West African countries.** *AIDS Behav* 2008, **12:**441-451.

40. Aho J, Nguyen VK, Diakité S, Sow A, Koushik A, Rashed S: **High acceptability of HIV voluntary counselling and testing among female sex workers: impact of individual and social factors.** *HIV Med* 2012, **13:**156-165.

41. Vuylsteke B, Vandenhoudt H, Langat L, Semde G, Menten J, Odongo F, Anapapa A, Sika L, Buve A, Laga M: **Capture-recapture for estimating the size of the female sex worker population in three cities in Côte d'Ivoire and in Kisumu, western Kenya.** *Trop Med Int Health* 2010, **15:**1537-1543.

42. Thomsen SC, Gallo MF, Ombidi W, Omungo Z, Janowitz B, Hawken M, Tucker H, Wong EL, Hobbs MM: **Randomised controlled trial on whether advance knowledge of prostate-specific antigen testing improves participant reporting of unprotected sex.** *Sex Transm Infect* 2007, **83:**419-420.

43. Gallo MF, Warner L, Bell AJ, Wiener J, Eschenbach DA, Bukusi EA, Sharma A, Njoroge B, Ngugi E, Jamieson DJ: **Assessment of changes in condom use among female sex workers in a prospective cohort study introducing diaphragm use for disease prevention.** *Am J Epidemiol* 2010, **172:**606-612.

44. Bukusi EA, Gallo MF, Sharma A, Njoroge B, Jamieson DJ, Nguti R, Bell AJ, Eschenbach DA: **Adherence to diaphragm use for infection prevention: a prospective study of female sex workers in Kenya.** *Infect Dis Obstet Gynecol* 2009, **2009:**420196.

45. Luchters S, Chersich MF, Rinyiru A, Barasa MS, King'ola N, Mandaliya K, Bosire W, Wambugu S, Mwarogo P, Temmerman M: **Impact of five years of peer-mediated interventions on sexual behavior and sexually transmitted infections among female sex workers in Mombasa, Kenya.** *BMC Public Health* 2008, **8:**143.

46. Njoroge B, Gallo MF, Sharma A, Bukusi EA, Nguti R, Bell AJ, Jamieson DJ, Williams D, Eschenbach DA: **Diaphragm for STI and HIV prevention: is it a safe method for women at high risk?** *Sex Transm Dis* 2010, **37:**382-385.

47. Luchters S, Chersich MF, Jao I, Schroth A, Chidagaya S, Mandaliya K, Temmerman M: **Acceptability of the diaphragm in Mombasa Kenya: a 6-month prospective study.** *Eur J Contracept Reprod Health Care* 2007, **12:**345-353.

48. Smith DJ, Wakasiaka S, Hoang TD, Bwayo JJ, Del Rio C, Priddy FH: **An evaluation of intravaginal rings as a potential HIV prevention device in urban Kenya: behaviors and attitudes that might influence uptake within a high-risk population.** *J Womens Health (Larchmt)* 2008, **17:**1025-1034.

49. Kreiss J, Ngugi E, Holmes K, Ndinya-Achola J, Waiyaki P, Roberts PL, Ruminjo I, Sajabi R, Kimata J, Fleming TR, et al.: **Efficacy of nonoxynol 9 contraceptive sponge use in preventing heterosexual acquisition of HIV in Nairobi prostitutes.** *JAMA* 1992, **268:**477-482.

50. Fonck K, Kaul R, Kimani J, Keli F, MacDonald KS, Ronald AR, Plummer FA, Kirui P, Bwayo JJ, Ngugi EN, et al: **A randomized, placebo-controlled trial of monthly azithromycin prophylaxis to prevent sexually transmitted infections and HIV-1 in Kenyan sex workers: study design and baseline findings.** *Int J STD AIDS* 2000, **11:**804-811.

51. Kaul R, Kimani J, Nagelkerke NJ, Fonck K, Ngugi EN, Keli F, MacDonald KS, Maclean IW, Bwayo JJ, Temmerman M, et al: **Monthly antibiotic chemoprophylaxis and incidence of sexually transmitted infections and HIV-1 infection in Kenyan sex workers: a randomized controlled trial.** *JAMA* 2004, **291:**2555-2562.

52. Yadav G, Saskin R, Ngugi E, Kimani J, Keli F, Fonck K, Macdonald KS, Bwayo JJ, Temmerman M, Moses S, Kaul R: **Associations of sexual risk taking among Kenyan female sex workers after enrollment in an HIV-1 prevention trial.** *J Acquir Immune Defic Syndr* 2005, **38:**329-334.

53. Kaul R, Nagelkerke NJ, Kimani J, Ngugi E, Bwayo JJ, Macdonald KS, Rebbaprgada A, Fonck K, Temmerman M, Ronald AR, Moses S: **Prevalent herpes simplex virus type 2 infection is associated with altered vaginal flora and an increased susceptibility to multiple sexually transmitted infections.** *J Infect Dis* 2007, **196:**1692-1697.

54. Ngugi EN, Chakkalackal M, Sharma A, Bukusi E, Njoroge B, Kimani J, MacDonald KS, Bwayo JJ, Cohen CR, Moses S, Kaul R: **Sustained changes in sexual behavior by female sex workers after completion of a randomized HIV prevention trial.** *J Acquir Immune Defic Syndr* 2007, **45:**588-594.

55. Ngugi EN, Wilson D, Sebstad J, Plummer FA, Moses S: **Focused peer-mediated educational programs among female sex workers to reduce sexually transmitted disease and human immunodeficiency virus transmission in Kenya and Zimbabwe.** *J Infect Dis* 1996, **174 Suppl 2:**S240-247.

56. Odek WO, Busza J, Morris CN, Cleland J, Ngugi EN, Ferguson AG: **Effects of micro-enterprise services on HIV risk behaviour among female sex workers in Kenya's urban slums.** *AIDS Behav* 2009, **13:**449-461.

57. Moses S, Plummer FA, Ngugi EN, Nagelkerke NJ, Anzala AO, Ndinya-Achola JO: **Controlling HIV in Africa: effectiveness and cost of an intervention in a high-frequency STD transmitter core group.** *AIDS* 1991, **5:**407-411.

58. Ngugi EN, Plummer FA, Simonsen JN, Cameron DW, Bosire M, Waiyaki P, Ronald AR, Ndinya-Achola JO: **Prevention of transmission of human immunodeficiency virus in Africa: effectiveness of condom promotion and health education among prostitutes.** *Lancet* 1988, **2:**887-890.

59. Ojoo J, Paul J, Batchelor B, Amir M, Kimari J, Mwachari C, Bwayo J, Plummer F, Gachihi G, Waiyaki P, Gilks C: **Bacteriuria in a cohort of predominantly HIV-1 seropositive female commercial sex workers in Nairobi, Kenya.** *J Infect* 1996, **33:**33-37.

60. Bandewar SV, Kimani J, Lavery JV: **The origins of a research community in the Majengo Observational Cohort Study, Nairobi, Kenya.** *BMC public health* 2010, **10:**630.

61. Witte K, Cameron KA, Lapinski MK, Nzyuko S: **A theoretically based evaluation of HIV/AIDS prevention campaigns along the trans-Africa highway in Kenya.** *J Health Commun* 1998, **3:**345-363.

62. Behets F, Turner AN, Van Damme K, Rabenja NL, Ravelomanana N, Zeller K, Rasolofomanana JR: **Acceptability and feasibility of continuous diaphragm use among sex workers in Madagascar.** *Sex Transm Infect* 2005, **81:**472-476.

63. Behets FM, Rasolofomanana JR, Van Damme K, Vaovola G, Andriamiadana J, Ranaivo A, McClamroch K, Dallabetta G, Van Dam J, Rasamilalao D, Rasamindra A: **Evidence-based treatment guidelines for sexually transmitted infections developed with and for female sex workers.** *Trop Med Int Health* 2003, **8:**251-258.

64. Feldblum PJ, Hatzell T, Van Damme K, Nasution M, Rasamindrakotroka A, Grey TW: **Results of a randomised trial of male condom promotion among Madagascar sex workers.** *Sex Transm Infect* 2005, **81:**166-173.

65. Hoke TH, Feldblum PJ, Van Damme K, Nasution MD, Grey TW, Wong EL, Ralimamonjy L, Raharimalala L, Rasamindrakotroka A: **Temporal trends in sexually transmitted infection prevalence and condom use following introduction of the female condom to Madagascar sex workers.** *Int J STD AIDS* 2007, **18:**461-466.

66. Pettifor AE, Turner AN, Van Damme K, Hatzell-Hoke T, Rasamindrakotroka A, Nasution MD, Behets F: **Increased risk of chlamydial and gonococcal infection in adolescent sex workers in Madagascar.** *Sex Transm Dis* 2007, **34:**475-478.

67. Hoke TH, Feldblum PJ, Damme KV, Nasution MD, Grey TW, Wong EL, Ralimamonjy L, Raharimalala L, Rasamindrakotroka A: **Randomised controlled trial of alternative male and female condom promotion strategies targeting sex workers in Madagascar.** *Sex Transm Infect* 2007, **83:**448-453.

68. Yotebieng M, Turner AN, Hoke TH, Van Damme K, Rasolofomanana JR, Behets F: **Effect of consistent condom use on 6-month prevalence of bacterial vaginosis varies by baseline BV status.** *Trop Med Int Health* 2009, **14:**480-486.

69. McClamroch KJ, Kaufman JS, Behets FM: **A formal decision analysis identifies an optimal treatment strategy in a resource-poor setting.** *J Clin Epidemiol* 2008, **61:**776-787.

70. Smith JS, Van Damme K, Randrianjafisamindrakotroka N, Ting J, Rabozakandraina T, Randrianasolo BS, Raharinivo M, Zanasaotra S, Hobbs M, Rinas A, et al: **Human papillomavirus and cervical neoplasia among female sex workers in Madagascar.** *Int J Gynecol Cancer* 2010, **20:**1593-1596.

71. Pettifor A, Turner AN, Swezey T, Khan M, Raharinivo MS, Randrianasolo B, Penman-Aguilar A, Van Damme K, Jamieson DJ, Behets F: **Perceived control over condom use among sex workers in Madagascar: a cohort study.** *BMC Womens Health* 2010, **10:**4.

72. Penman-Aguilar A, Legardy-Williams J, Turner AN, Rabozakandriana TO, Williams D, Razafindravoavy S, Behets F, Van Damme K, Jamieson DJ: **Effect of treatment assignment on intravaginal cleansing in a randomized study of the diaphragm with candidate microbicide.** *J Womens Health (Larchmt)* 2011, **20:**187-195.

73. Feldblum PJ, Nasution MD, Hoke TH, Van Damme K, Turner AN, Gmach R, Wong EL, Behets F: **Pregnancy among sex workers participating in a condom intervention trial highlights the need for dual protection.** *Contraception* 2007, **76:**105-110.

74. McClamroch K, Behets F, Van Damme K, Rabenja LN, Myers E: **Cost-effectiveness of treatment strategies for cervical infection among women at high risk in Madagascar.** *Sex Transm Dis* 2007, **34:**631-637.

75. Kruse N, Behets FM, Vaovola G, Burkhardt G, Barivelo T, Amida X, Dallabetta G: **Participatory mapping of sex trade and enumeration of sex workers using capture-recapture methodology in Diego-Suarez, Madagascar.** *Sex Transm Dis* 2003, **30:**664-670.

76. Zachariah R, Harries AD, Buhendwa L, Spielman MP, Chantulo A, Bakali E: **Acceptability and technical problems of the female condom amongst commercial sex workers in a rural district of Malawi.** *Trop Doct* 2003, **33:**220-224.

77. Zachariah R, Spielmann MP, Harries AD, Nkhoma W, Chantulo A, Arendt V: **Sexually transmitted infections and sexual behaviour among commercial sex workers in a rural district of Malawi.** *Int J STD AIDS* 2003, **14:**185-188.

78. Walden VM, Mwangulube K, Makhumula-Nkhoma P: **Measuring the impact of a behaviour change intervention for commercial sex workers and their potential clients in Malawi.** *Health Educ Res* 1999, **14:**545-554.

79. Kalanda B: **Empowering young sex workers for safer sex in Dowa and Lilongwe Districts of Malawi.** *Malawi Med J* 2010, **22:**10-11.

80. Lafort Y, Geelhoed D, Cumba L, Lazaro CD, Delva W, Luchters S, Temmerman M: **Reproductive health services for populations at high risk of HIV: Performance of a night clinic in Tete province, Mozambique.** *BMC Health Serv Res* 2010, **10:**144.

81. Agha S, Chulu Nchima M: **Life-circumstances, working conditions and HIV risk among street and nightclub-based sex workers in Lusaka, Zambia.** *Cult Health Sex* 2004, **6:**283-299.

82. Agha S, Karlyn A, Meekers D: **The promotion of condom use in non-regular sexual partnerships in urban Mozambique.** *Health Policy Plan* 2001, **16:**144-151.

83. Fitzgerald-Husek A, Martiniuk AL, Hinchcliff R, Aochamus CE, Lee RB: **"I do what I have to do to survive": an investigation into the perceptions, experiences and economic considerations of women engaged in sex work in Northern Namibia.** *BMC Womens Health* 2011, **11:**35.

84. Esu-Williams E, Phillips, A. L. and Githens, W.: **AIDS Prevention: A Guide for Working with Commercial Sex Workers. Experiences from Calabar, Nigeria**In *Book AIDS Prevention: A Guide for Working with Commercial Sex Workers. Experiences from Calabar, Nigeria*(Editor ed.^eds.). City; 1993.

85. Imade G, Sagay A, Egah D, Onwuliri V, Grigg M, Egbodo C, Thacher T, Potts M, Short R: **Prevalence of HIV and other sexually transmissible infections in relation to lemon or lime juice douching among female sex workers in Jos, Nigeria.** *Sex Health* 2008, **5:**55-60.

86. Braunstein SL, Ingabire CM, Geubbels E, Vyankandondera J, Umulisa MM, Gahiro E, Uwineza M, Tuijn CJ, Nash D, van de Wijgert JH: **High burden of prevalent and recently acquired HIV among female sex workers and female HIV voluntary testing center clients in Kigali, Rwanda.** *PloS one* 2011, **6:**e24321.

87. Braunstein SL, Ingabire CM, Kestelyn E, Uwizera AU, Mwamarangwe L, Ntirushwa J, Nash D, Veldhuijzen NJ, Nel A, Vyankandondera J, van de Wijgert JH: **High human immunodeficiency virus incidence in a cohort of Rwandan female sex workers.** *Sex Transm Dis* 2011, **38:**385-394.

88. Braunstein SL, Nash D, Kim AA, Ford K, Mwambarangwe L, Ingabire CM, Vyankandondera J, van de Wijgert JH: **Dual testing algorithm of BED-CEIA and AxSYM Avidity Index assays performs best in identifying recent HIV infection in a sample of Rwandan sex workers.** *PLoS One* 2011, **6:**e18402.

89. Braunstein SL, Umulisa MM, Veldhuijzen NJ, Kestelyn E, Ingabire CM, Nyinawabega J, van de Wijgert JH, Nash D: **HIV diagnosis, linkage to HIV care, and HIV risk behaviors among newly diagnosed HIV-positive female sex workers in Kigali, Rwanda.** *J Acquir Immune Defic Syndr* 2011, **57:**e70-76.

90. Leonard L, Ndiaye I, Kapadia A, Eisen G, Diop O, Mboup S, Kanki P: **HIV prevention among male clients of female sex workers in Kaolack, Senegal: results of a peer education program.** *AIDS Educ Prev* 2000, **12:**21-37.

91. Wang C, Hawes SE, Gaye A, Sow PS, Ndoye I, Manhart LE, Wald A, Critchlow CW, Kiviat NB: **HIV prevalence, previous HIV testing, and condom use with clients and regular partners among Senegalese commercial sex workers.** *Sex Transm Infect* 2007, **83:**534-540.

92. Laurent C, Seck K, Coumba N, Kane T, Samb N, Wade A, Liégeois F, Mboup S, Ndoye I, Delaporte E: **Prevalence of HIV and other sexually transmitted infections, and risk behaviours in unregistered sex workers in Dakar, Senegal.** *AIDS* 2003, **17:**1811-1816.

93. Pettifor AE, Beksinska ME, Rees HV, Mqoqi N, Dickson-Tetteh KE: **The acceptability of reuse of the female condom among urban South African women.** *J Urban Health* 2001, **78:**647-657.

94. Richter M, Yarrow J, Delany-Moretlwe S: **The women-at-risk project: providing tailored health care to sex workers in inner-city Johannesburg, South Africa.** In *Book The women-at-risk project: providing tailored health care to sex workers in inner-city Johannesburg, South Africa* (Editor ed.^eds.). City; 2008.

95. Vickerman P, Terris-Prestholt F, Delany S, Kumaranayake L, Rees H, Watts C: **Are targeted HIV prevention activities cost-effective in high prevalence settings? Results from a sexually transmitted infection treatment project for sex workers in Johannesburg, South Africa.** *Sex Transm Dis* 2006, **33:**S122-132.

96. Stadler J, Delany S: **The 'healthy brothel': the context of clinical services for sex workers in Hillbrow, South Africa.** *Cult Health Sex* 2006, **8:**451-464.

97. Dunkle KL, Beksinska ME, Rees VH, Ballard RC, Htun Y, Wilson ML: **Risk factors for HIV infection among sex workers in Johannesburg, South Africa.** *Int J STD AIDS* 2005, **16:**256-261.

98. Pauw I, Brener L: **'You are just whores - you can't be raped': Barriers to safer sex practices among women street sex workers in Cape Town.** *Culture, Health & Sexuality* 2003, **5:**465-481.

99. Williams BG, Taljaard D, Campbell CM, Gouws E, Ndhlovu L, Van Dam J, Carael M, Auvert B: **Changing patterns of knowledge, reported behaviour and sexually transmitted infections in a South African gold mining community.** *Aids* 2003, **17:**2099-2107.

100. Connolly CA, Ramjee G, Sturm AW, Abdool Karim SS: **Incidence of Sexually Transmitted Infections among HIV-positive sex workers in KwaZulu-Natal, South Africa.** *Sex Transm Dis* 2002, **29:**721-724.

101. Ramjee G, Gouws E: **Prevalence of HIV among truck drivers visiting sex workers in KwaZulu-Natal, South Africa.** *Sex Transm Dis* 2002, **29:**44-49.

102. Ramjee G, Williams B, Gouws E, Van Dyck E, De Deken B, Karim SA: **The impact of incident and prevalent herpes simplex virus-2 infection on the incidence of HIV-1 infection among commercial sex workers in South Africa.** *J Acquir Immune Defic Syndr* 2005, **39:**333-339.

103. Ramjee G, Karim SS, Sturm AW: **Sexually transmitted infections among sex workers in KwaZulu-Natal, South Africa.** *Sex Transm Dis* 1998, **25:**346-349.

104. Karim QA, Karim SS, Soldan K, Zondi M: **Reducing the risk of HIV infection among South African sex workers: socioeconomic and gender barriers.** *Am J Public Health* 1995, **85:**1521-1525.

105. Auvert B, Marais D, Lissouba P, Zarca K, Ramjee G, Williamson AL: **High-risk human papillomavirus is associated with HIV acquisition among South African female sex workers.** *Infect Dis Obstet Gynecol* 2011, **2011:**692012.

106. Bures R, Morris L, Williamson C, Ramjee G, Deers M, Fiscus SA, Abdool-Karim S, Montefiori DC: **Regional clustering of shared neutralization determinants on primary isolates of clade C human immunodeficiency virus type 1 from South Africa.** *J Virol* 2002, **76:**2233-2244.

107. Rustomjee R, Abdool Karim Q, Abdool Karim SS, Laga M, Stein Z: **Phase 1 trial of nonoxynol-9 film among sex workers in South Africa.** *AIDS* 1999, **13:**1511-1515.

108. Vandebosch A, Goetghebeur E, Ramjee G, Alary M, Ettiegne-Traore V, Chandeying V, Van Damme L: **Acceptability of COL-1492, a vaginal gel, among sex workers in one Asian and three African cities.** *Sex Transm Infect* 2004, **80:**241-243.

109. Steen R, Dallabetta G: **The use of epidemiologic mass treatment and syndrome management for sexually transmitted disease control.** *Sex Transm Dis* 1999, **26:**S12-20; discussion S21-12.

110. Steen R, Vuylsteke B, DeCoito T, Ralepeli S, Fehler G, Conley J, Bruckers L, Dallabetta G, Ballard R: **Evidence of declining STD prevalence in a South African mining community following a core-group intervention.** *Sex Transm Dis* 2000, **27:**1-8.

111. Makinwa B, O'Grady M: **FHI/UNAIDS Best Practices in HIV Prevention Collection.** In *Book FHI/UNAIDS Best Practices in HIV Prevention Collection* (Editor ed.^eds.). City: Family Health International; 2001.

112. Marseille E, Kahn JG, Billinghurst K, Saba J: **Cost-effectiveness of the female condom in preventing HIV and STDs in commercial sex workers in rural South Africa.** *Soc Sci Med* 2001, **52:**135-148.

113. Wechsberg WM, Luseno WK, Lam WK: **Violence against substance-abusing South African sex workers: intersection with culture and HIV risk.** *AIDS Care* 2005, **17 Suppl 1:**S55-64.

114. Wechsberg WM, Luseno WK, Lam WK, Parry CD, Morojele NK: **Substance use, sexual risk, and violence: HIV prevention intervention with sex workers in Pretoria.** *AIDS Behav* 2006, **10:**131-137.

115. Wilson D: ***Corridors of Hope in Southern Africa: HIV Prevention Needs and Opportunities in Four Border Towns*.** In *Book Corridors of Hope in Southern Africa: HIV Prevention Needs and Opportunities in Four Border Towns* (Editor ed.^eds.). City: Family Health International, USAID; 2005.

116. Nyamuryekung'e K, Laukamm-Josten U, Vuylsteke B, Mbuya C, Hamelmann C, Outwater A, Steen R, Ocheng D, Msauka A, Dallabetta G: **STD services for women at truck stop in Tanzania: evaluation of acceptable approaches.** *East Afr Med J* 1997, **74:**343-347.

117. Sobéla F, Pépin J, Gbéléou S, Banla AK, Pitche VP, Adom W, Sodji D, Frost E, Deslandes S, Labbé AC: **A tale of two countries: HIV among core groups in Togo.** *J Acquir Immune Defic Syndr* 2009, **51:**216-223.

118. Vandepitte J, Bukenya J, Weiss HA, Nakubulwa S, Francis SC, Hughes P, Hayes R, Grosskurth H: **HIV and other sexually transmitted infections in a cohort of women involved in high-risk sexual behavior in Kampala, Uganda.** *Sex Transm Dis* 2011, **38:**316-323.

119. **The World Bank HIV/AIDS Project For Abidjan/Lagos Transport Corridor: Implementation completion and results report**In *Book The World Bank HIV/AIDS Project For Abidjan/Lagos Transport Corridor: Implementation completion and results report*(Editor ed.^eds.). City: The World Bank; 2008.

120. Morin D, Godin G, Alary M, Sawadogo MR, Bernier M, Khonde N, Kintin F, Kone A, N'Dour M, Pepin J, et al: **Satisfaction with health services for STIs, HIV, AIDS among a high-risk population in West Africa.** *AIDS Care* 2008, **20:**388-394.

121. Pépin J, Sobela F, Khonde N, Agyarko-Poku T, Diakité S, Deslandes S, Labbé AC, Sylla M, Asamoah-Adu C, Frost E: **The syndromic management of vaginal discharge using single-dose treatments: a randomized controlled trial in West Africa.** *Bull World Health Organ* 2006, **84:**729-738.

122. Jain S, Greene M, Douglas Z, Betron M, Fritz K: ***Risky Business Made Safer. Corridors of Hope: An HIV Prevention Program in Zambian Border and Transit Towns***In *Book Risky Business Made Safer. Corridors of Hope: An HIV Prevention Program in Zambian Border and Transit Towns*(Editor ed.^eds.). City: USAID; 2011.

123. Wilson D, Sibanda B, Mboyi L, Msimanga S, Dube G: **A pilot study for an HIV prevention programme among commercial sex workers in Bulawayo, Zimbabwe.** *Soc Sci Med* 1990, **31:**609-618.

124. Chipfakacha V: **Prevention of sexually transmitted disease. The Shurugwi sex-workers project.** *S Afr Med J* 1993, **83:**40-41.
